# Lead generation of UPPS inhibitors targeting MRSA: Using 3D-QSAR pharmacophore modeling, virtual screening, molecular docking, and molecular dynamic simulations

**DOI:** 10.1186/s13065-023-01110-1

**Published:** 2024-01-20

**Authors:** Basma M. Qandeel, Samar Mowafy, Khaled Abouzid, Nahla A. Farag

**Affiliations:** 1 Pharmaceutical Chemistry Department, Faculty of Pharmacy, Misr International University, Km28 Cairo-Ismailia Road, Ahmed Orabi District, Cairo, Egypt; 2 Department of Pharmaceutical Chemistry, College of Pharmacy, Ain-Shams University, Abbasia, 11566 Egypt

**Keywords:** Antibacterial, UPPS, Methicillin-resistant *Staphylococcus aureus*, Bacterial resistance, HypoGen algorithm, Molecular docking, 3D QSAR pharmacophore, Dynamic simulations, Drug repurposing

## Abstract

Undecaprenyl Pyrophosphate Synthase (UPPS) is a vital target enzyme in the early stages of bacterial cell wall biosynthesis. UPPS inhibitors have antibacterial activity against resistant strains such as MRSA and VRE. In this study, we used several consecutive computer-based protocols to identify novel UPPS inhibitors. The 3D QSAR pharmacophore model generation (HypoGen algorithm) protocol was used to generate a valid predictive pharmacophore model using a set of UPPS inhibitors with known reported activity. The developed model consists of four pharmacophoric features: one hydrogen bond acceptor, two hydrophobic, and one aromatic ring. It had a correlation coefficient of 0.86 and a null cost difference of 191.39, reflecting its high predictive power. Hypo1 was proven to be statistically significant using Fischer’s randomization at a 95% confidence level. The validated pharmacophore model was used for the virtual screening of several databases. The resulting hits were filtered using SMART and Lipinski filters. The hits were docked into the binding site of the UPPS protein, affording 70 hits with higher docking affinities than the reference compound (6TC, − 21.17 kcal/mol). The top five hits were selected through extensive docking analysis and visual inspection based on docking affinities, fit values, and key residue interactions with the UPPS receptor. Moreover, molecular dynamic simulations of the top hits were performed to confirm the stability of the protein–ligand complexes, yielding five promising novel UPPS inhibitors.

**Graphical Abstract:**

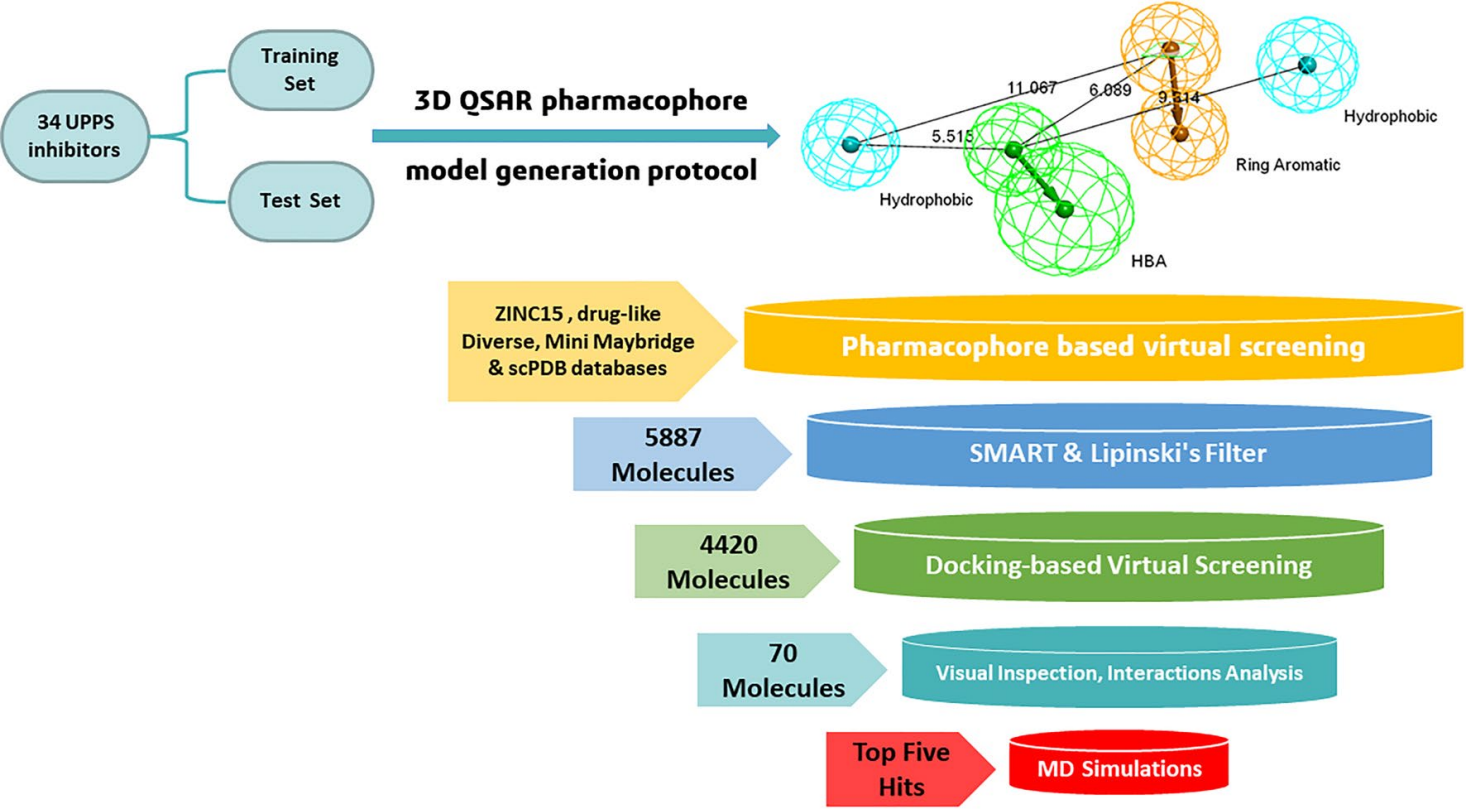

**Supplementary Information:**

The online version contains supplementary material available at 10.1186/s13065-023-01110-1.

## Introduction

Due to the threat of emerging antibiotic resistance, the quest for new antibacterial agents remains an essential endeavor in drug discovery. Many antibacterial agents, such as methicillin and vancomycin, display a bactericidal effect by inhibiting bacterial cell wall synthesis. However, Methicillin-Resistant *Staphylococcus Aureus* (MRSA) and Vancomycin-Resistant *Enterococci* (VRE) are emerging and pose a major threat [1–4]. One strategy for overcoming bacterial resistance to most cell wall synthesis-inhibiting antibiotics is to utilize inhibitors that target different enzymes within the same pathway as current antibiotics [5]. This approach can help avoid cross-resistance development, create a synergistic effect, and possibly restore sensitivity through combination therapy [5–7].

**Fig. 1:**
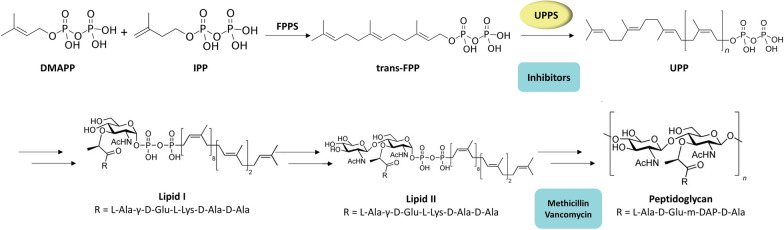
Biosynthetic pathway of peptidoglycan bacterial cell wall including sites of action of UPPS inhibitors, methicillin, and vancomycin

One such enzyme is Undecaprenyl Pyrophosphate Synthase (UPPS), an integral target enzyme in the early steps of bacterial cell wall biosynthesis. Undecaprenyl Pyrophosphate Synthase is part of the family of cis-prenyltransferases [8]. UPPS catalyzes the continuous condensation of eight molecules of Isopentenyl Pyrophosphate (IPP) with Farnesyl Pyrophosphate (FPP), producing C55 Undecaprenyl Pyrophosphate (UPP) [9]. Then C55-isoprenol pyrophosphate phosphatase removes the terminal phosphate of UPP, forming Undecaprenyl Phosphate (UP) (Fig. 1) [5], an imperative anchor for the synthesis of lipid I and lipid II and the assembly of the peptidoglycan cell wall [5, 10–13]. UPPS is an attractive target because it is essential for bacterial cell growth while absent from humans [14]. Nonetheless, UPPS inhibitors possess antibacterial activity on resistant strains such as MRSA and VRE when used alone or in combination with current agents [15–18].

While UPPS is a validated target, no selective inhibitors have been reported in the literature. In vitro activity and bioavailability of the substrate analogs reported were modest, highlighting the need for novel, more effective antibacterial agents that target UPPS [19–22]. Most small-molecule UPPS inhibitors are highly hydrophobic compounds containing ionic groups such as bisphosphonates and high molecular weight spirohexalines [16]. Most of these compounds are poorly absorbed and exhibit modest in vitro activity, making their selectivity and suitability for antimicrobial drug design questionable [22–24]. This research aims to identify novel, more effective antibacterial agents that target UPPS.

Various crystal structures of the UPPS protein have been reported, demonstrating its flexibility in its native, substrate-bound, and product-bound states [25–27]. A druggable active site and an essential role in the cell wall synthesis of many pathogenic bacteria make UPPS an attractive target for new antibacterial drugs [28, 29]. Consequently, virtual and high-throughput screenings were conducted to find inhibitors of UPPS that are not bisphosphonates and possess antimicrobial activities against clinically relevant strains [21, 22, 27, 30–32]. Most of these computer-aided drug design approaches towards discovering new non-bisphosphonates UPPS inhibitors relied solely on in silico target-based virtual screening of large libraries of compounds without using filters to ensure the drug likeability of the hit compounds [5, 14, 15, 33, 34]. While these approaches yielded the discovery of some UPPS inhibitors, they hardly provide any structure–activity relationships [33].

Here, we report using consecutive computer-aided drug design protocols, including 3D QSAR pharmacophore generation, in silico virtual screening, docking, and molecular dynamics to identify novel potential UPPS inhibitors [35]. First, we performed ligand-based 3D QSAR pharmacophore generation using a data set library of 25 UPPS inhibitors synthesized and reported by Novartis. The ligands belong to a dataset of tetramic and tetronic acids with IC_50_ in the 100-nM range and dihydropyridines with IC_50_ down to 40 nM, all with antibacterial activity against Gram-positive bacteria [36]. The HypoGen algorithm summarized the structural features of these ligands to generate a valid predictive pharmacophore model using the Discovery Studio V4.1 software package [37]. The correlation coefficient between the predicted and experimental activities was 0.8699 for the training set and 0.8177 for the test set, thus indicating good predictive ability. The chosen pharmacophore model (Hypo 1) was further validated using cost analysis and Fischer’s randomization. The valid pharmacophore model was used to virtually screen several databases, such as FDA-approved molecules from the ZINC15 library, Drug-Like Diverse, Mini Maybridge, and scPDB. Subsequent filtration was done to assess the drug-likeability of the hits. Three conditions were applied: (a) Lipinski’s Rules of Five, which assessed the drug-likeability of the compounds, (b) SMART filtration, which eliminated unneeded functional groups and (c) Filtration criteria limited to fit values above 6.5. The virtual screening hits were docked via the CDOCKER protocol into the binding site of the crystal structure of *Streptococcus pneumoniae* Undecaprenyl Pyrophosphate Synthase (UPPS) (PDB ID: 5KH5) [38] in complex with the pyrazole inhibitor *N*-(3-amino-3-isopropyl)-5-(benzo[b]thiophen-6-yl)-1-benzyl-*N*-(4-isopropoxy phenyl)-1*H*-pyrazole-4-carboxamide (**6TC**). After analyzing the docking scores and pharmacophore fit values, potential active candidates that target UPPS were identified. Molecular dynamic simulations of the top hit-protein complexes were performed to validate the docking results and confirm the stability of the protein–ligand complexes.

## Materials and methods

Ligand-based and structure-based computer-aided drug design are used to discover new drug leads by employing ligands with known activity to help develop new biologically active leads for a specific target. The traditional ways of developing new drugs are inefficient in cost, time, and effort. In contrast, computer-aided drug design allows us to make better-informed decisions, hence exhausting fewer resources [39–41].

In this endeavor, the 3D QSAR Pharmacophore generation protocol (DS) was used to develop new antibacterial leads targeting the UPPS enzyme. To generate the primary data set for the 3D QSAR pharmacophore modeling study, 34 molecules with an excellent range of inhibitory activity on UPPS enzyme were extracted from previously published literature [36, 42]. The experimental inhibitory activity of all the 34 ligands included in the datasets was acquired via the same bioassays on streptococcal UPPS enzyme with IC_50_ values ranging from 0.04 to 58 μM.

The dataset was divided into training and test sets based on the following criteria to achieve a significant pharmacophore model: (1) The dataset was categorized into four activity levels: highly active (IC_50_ ≤ 0.1 μM), active (IC_50_: 0.1–1.0 μM), moderately active (IC_50_: 1.0–10.0 μM), and inactive (IC_50_ > 10) [43]. (2) A diverse distribution of the four biological activity levels was ensured in both training and test sets, with the training test including a maximum number of highly active and active compounds and some of the moderately active and inactive compounds while the remaining compounds were assigned to the test set for validation [44]. (3) Both sets include diverse chemical derivatizations of dihydropyridines, tetramic, and tetronic acids [43].

**Fig. 2:**
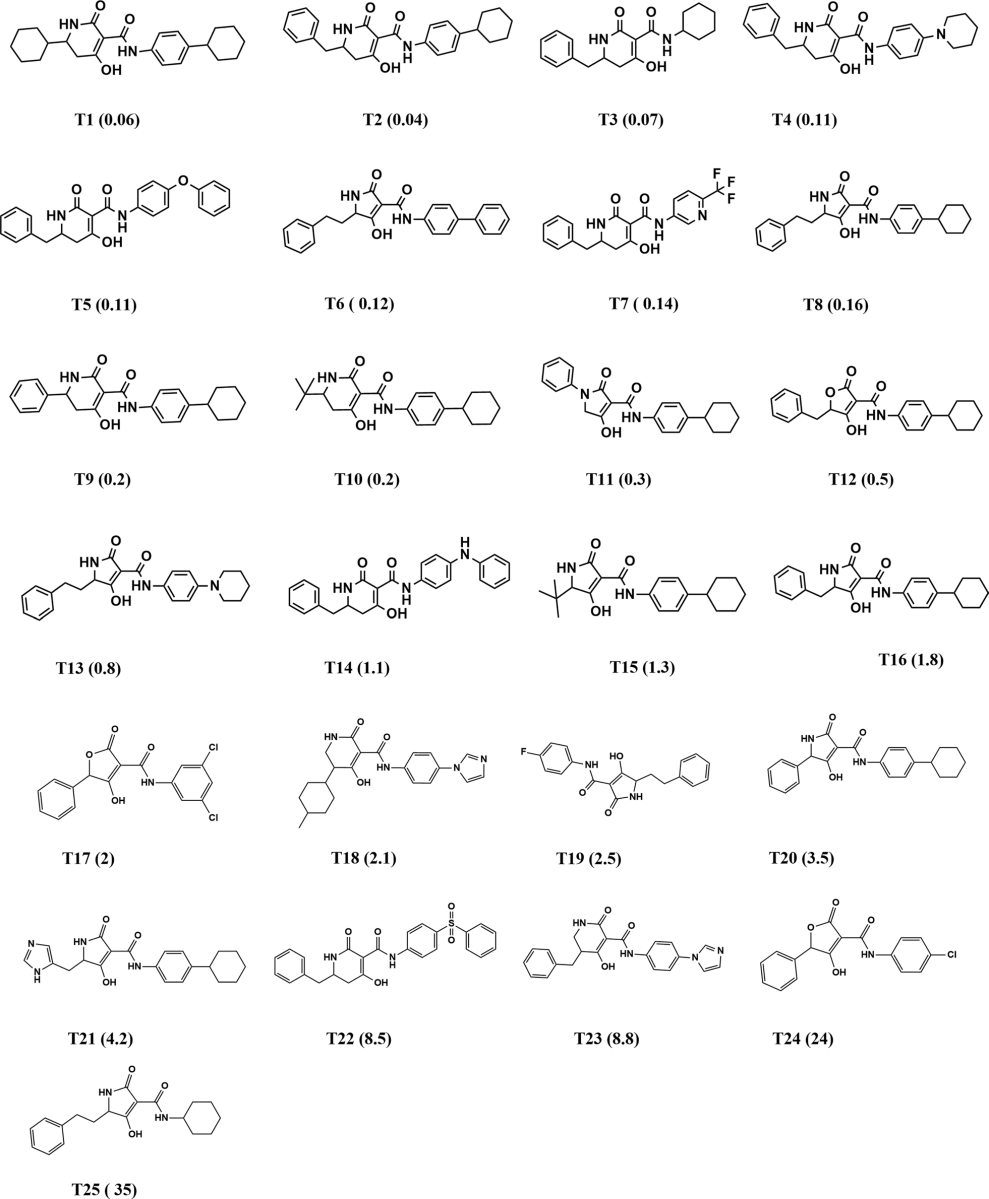
Training set ligands along with their IC_50_ values ranging from 0.04 to 35 μM

**Fig. 3:**
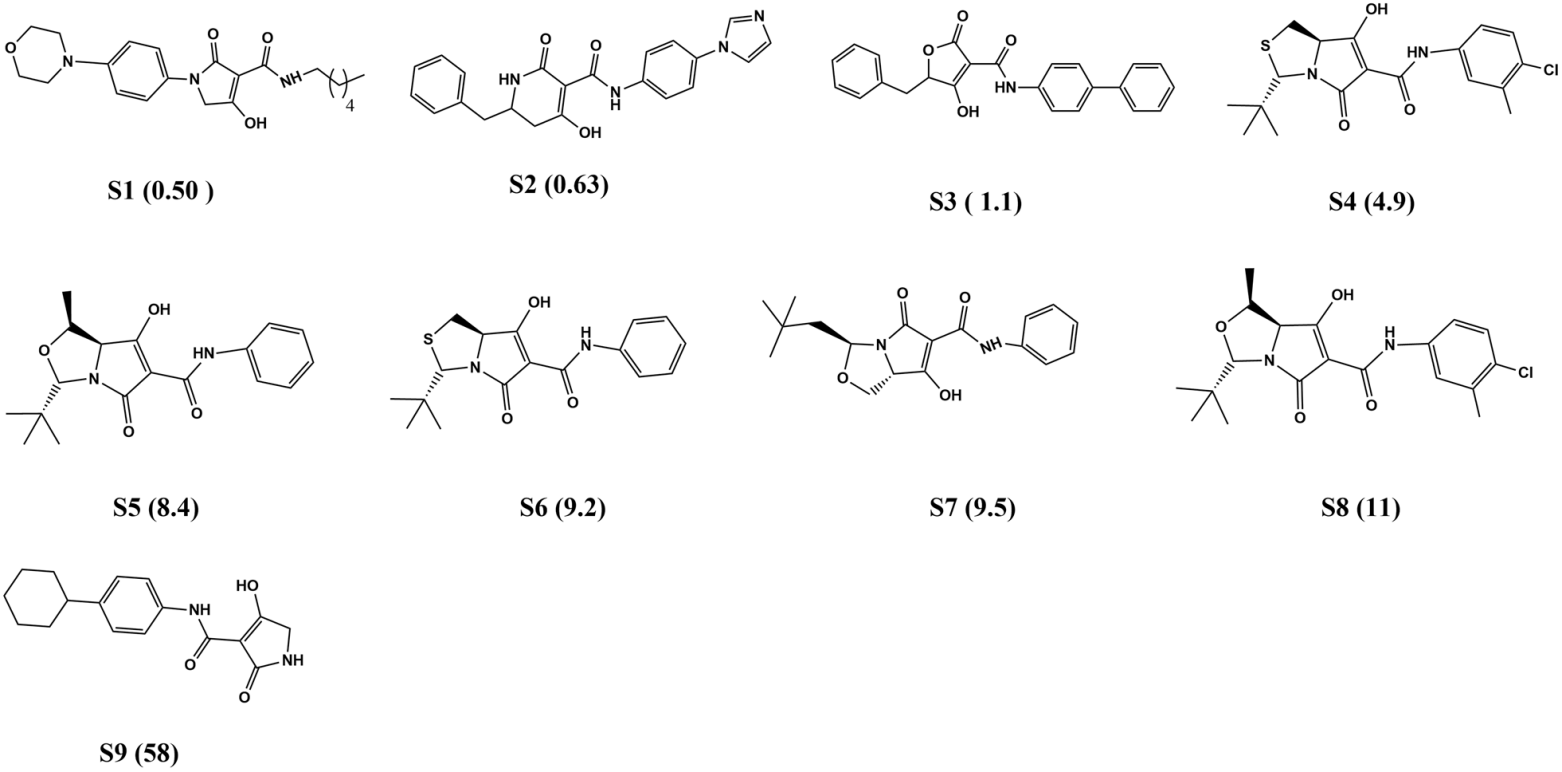
Test set ligands along with their IC_50_ values ranging from 0.5 μM to 58 μM

Finally, after following the abovementioned criteria, 25 diversified ligands were considered in the training set (Fig. 2), with experimental IC_50_ values ranging from 0.04 to 35 μM [36]. Nine compounds were assigned to the test set (Fig. 3), with experimental IC_50_ values ranging from 0.5 to 58 μM [36, 42].

### Compounds preparation

The two-dimensional (2D) structures of the datasets were drawn using ChemDraw Ultra and subsequently converted into their three-dimensional (3D) form by Discovery Studio 4.1 (DS). The datasets were prepared using the prepare ligands protocol of (DS). This protocol prepares ligands for input into other protocols, executing tasks such as removing duplicates, enumerating isomers and tautomers, and generating 3D conformations. The prepare ligand protocol provides reasonable starting ligand structures to achieve good results in the subsequent protocol. Additionally, it enumerates valid ionization states and compounds with undesirable properties. This protocol accomplishes these tasks by performing the following steps: (1) Generating canonical tautomers, (2) Keeping only the largest fragments, (3) Setting standard formal charges on common functional groups, (4) Kekulizing molecules, which is assigning double bonds to the molecular graph using DS as a guide before assigning virtual hydrogen, (5) Fixing bad valences, (6) Generating a reasonable 3D conformation.

Furthermore, the generate conformations protocol in DS was utilized to create optimized conformations for the training set and test set. The CHARMM force field was used to achieve energy-minimized conformations of each compound in the training and test sets. Throughout the conformation’s generation process, parameters such as the maximum number of conformers were set to 255, and the energy threshold was set to 20 kcal/mol. These conformers were used to generate pharmacophore hypotheses, fit the ligands into the model hypothesis, and predict the activity of newly investigated compounds [45, 46].

### Pharmacophore model generation

3D QSAR pharmacophore modeling is a ligand-based computer-aided drug design (CADD) method. The protocol utilizes the chemical properties of a dataset of diverse ligands with a broad range of biological activity on a specific target enzyme. This is done to design a valid predictive pharmacophore that reflects the necessary chemical features responsible for biological activity. The generated pharmacophore can be used to identify new candidates and predict their biological activity [47–49].

In this endeavor, a training set of 25 known active UPPS inhibitors with a wide range of activity represented in IC_50_ is used to create a pharmacophore model. The training set was subjected to the feature mapping protocol in DS to identify distinct chemical features present in the ligands. The features revealed were Hydrogen Bond Acceptor (HBA), Hydrogen Bond Donor (HBD), Hydrophobic (HYD), and Ring Aromatic (RA). The four features identified were chosen for the 3D QSAR pharmacophore generation protocol. The IC_50_ was selected to be the active property, and the energy threshold was retained at 20 kcal/mol throughout the protocol run. The uncertainty value was set to 1.5. This value represents a ratio of the reported value to the minimum and maximum values. Setting the uncertainty value to 1.5 entails that the model can acclimate differences in the experimental IC_50_ values and predict IC_50_ up to 1.5 times [44]. All the other parameters were left to default.

The HypoGen algorithm utilized in the 3D QSAR pharmacophore generation protocol of DS interpreted the common chemical features related to low or high biological activity in the training set. The pharmacophore model utilized in this work was chosen out of 10 generated hypotheses according to possessing the highest correlation coefficient, lowest total cost, and Root Mean Square (RMS).

### Pharmacophore model validation

The pharmacophore model was validated via three evident means: test set analysis, cost analysis, and Fischer’s randomization.

In test set analysis, the ligand pharmacophore mapping protocol in DS overlaps the selected pharmacophore with a test of ligands with varying experimental activity, thus providing estimated activities of the test set (Table 4). The closer the estimated activities are to the experimental activities of the test set, the more predictive the pharmacophore is. An acceptable correlation coefficient with a cross-validation 95% confidence level should be attained to consider a pharmacophore model predictive [44, 50]

The HYPOGEN algorithm in the Discovery Studio software calculates various cost functions and correlation values that can be interpreted to validate a given pharmacophore. The fixed cost assumes a simple hypothesis model that seamlessly fits all the dataset molecules; hence, it is the lowest cost [51]. The fixed cost value for the generated hypothesis is 74.40. The null cost, on the other hand, is equivalent to the highest possible error cost [52]. The null cost for the generated hypotheses is 332.267. The total cost is calculated independently for each pharmacophore hypothesis. It is the sum of weight, error, and fixed costs [44]. The total costs for the ten generated hypotheses ranged from 140 to 197. To consider a pharmacophore model robust, the total cost value of the evaluated pharmacophore should be close to the fixed cost and distant from the null cost. The best model was selected based on the null cost distance; a null cost distance value of more than 60 indicates a significant correlation and denotes that the model is > 90% accurate in the prediction of activity [53]

Fischer’s randomization validation technique allows us to evaluate the statistical significance of the hypotheses generated by the HypoGen algorithm via statistical validation [44]. A 95% confidence level was selected, and the training set ligands were randomly given activity values and allowed to generate 19 random spreadsheets (random hypotheses). For the pharmacophore generation process to be valid, the ten pharmacophore hypotheses generated by HypoGen should have superior total cost values and statistically significant correlations compared to the 19 random spreadsheets created by Fischer’s randomization [54, 55].

### Virtual screening

Virtual screening (VS) is a drug discovery strategy that searches libraries of small molecules for structures with the highest probability of binding to a drug target [56]. Based on the generated pharmacophore, a virtual screening was initiated to identify structurally novel and potentially active UPPS inhibitors from diverse chemical databases. Hypo 1 was allowed to screen 32387 molecules belonging to FDA-approved molecules from the ZINC15 [57], drug-like Diverse, MiniMaybridge, and scPDB libraries. Hit molecules should fit into all the chemical features of Hypo 1. The Search 3D database protocol was utilized with the search option set to best/flexible to obtain promising hit molecules from the database. Compounds with high fit values (close to the fit value of the reference **6TC**) were subjected to various constraints to refine the hits further. Constraints like Lipinski’s and SMART filters were applied to ensure the drug-likeability of the selected hits. Lipinski’s rule of five is an important filter that considers the molecules’ pharmacokinetics to ensure that the chosen molecules can be absorbed orally and have drug-like molecular properties [58, 59]. On the other hand, the SMART filter removes molecules with toxic functional groups like sulfonyl halide, sulfonate ester, cyanide, peroxide, and other functional groups that decrease the molecule’s drug-likeability [60, 61]. Finally, the filtered hits were docked onto the binding site of the target UPPS protein (PDB ID: 5KH5) [38], and docking scores, along with interactions, were used to identify the top hits.

### Molecular docking studies

All the molecular docking studies done were based on the crystal structure of *Streptococcus pneumoniae* Undecaprenyl pyrophosphate Synthase (UPPS) (PDB ID: 5KH5) [38] in complex with the pyrazole inhibitor **6TC** as the co-crystallized ligand. According to the literature and upon studying the crystal structure of UPPS (PDB ID: 5KH5) in complexes with the inhibitor, it is established that the active site of the protein has two neighboring pockets where the natural substrates FPP and IPP bind. The complex of UPPS bound to the natural substrate FPP (PDB ID: 5KH4) [38] shows the pyrophosphate directly interacts with the amino acids **Arg41** and **Arg79**, and the farnesyl tail binds into a long, deep cavity lined by several hydrophobic side chains. In the crystal structure of UPPS in complex with inhibitor (**6TC**) (PDB ID: 5KH5), the inhibitor **6TC** binds near the base of the hydrophobic pocket of the FPP binding site. Most of the interactions made by **6TC** are hydrophobic, along with two pi-stacking interactions between the benzyl-isopropyl ether moiety and **Phe143** and between the benzothiophene moiety and both **Phe94** and **Met49** [38].

Molecular docking was done via the CDOCKER protocol DS. The CDOCKER protocol allows us to simulate the docking of a ligand into the target’s binding site and utilizes several scoring functions to assess the docked poses [62]. A CHARMM-based molecular dynamics (MD) is used to dock ligands into the target protein’s active site, and high-temperature molecular dynamics generate random ligand conformers and translate them into the binding site [63]. The ligand conformers are developed through random rigid-body rotations followed by simulated annealing [64]. It has been demonstrated that the CHARMM-based C-docker protocol yields highly accurate docked poses [65]. The protein preparation tool was used to correct common problems in the protein structure by adding missing loops and hydrogens and excluding alternate conformers. The binding site was then identified by using the define and edit binding site tool, which resulted in a sphere of 8.2 Å from the geometric centroid of the co-crystallized ligand **6TC** and the binding site atomic coordinates were − 3.649150, 9.898302, and − 6.074069.

The ligand preparation tool prepared the selected hits from the pharmacophore-based virtual screening to fix incorrect valences and generate 3D conformers. Then, the hits were docked onto the defined binding site of the protein. The docking results were analyzed according to the CHARMM energy scoring function, the CDOCKER energy. To identify more desirable hit molecules, molecules with high docking scores that display interactions with the key active site residues and similar geometry as the reference **6TC** were selected (Tables 5 and 6).

### Induced fit docking

An induced fit docking (IFD) study was done using the Schrödinger package to confirm the docking results using the CDOCKER protocol. The top five hits selected from the docking studies and the reference compound 6TC were chosen for the IFD study. The IFD procedure includes three steps: (1) Selected hits were docked into a rigid receptor active site pocket. (2) A 0.5 van der Waals (VdW) scaling factor was applied to the protein and ligand’s non-polar atoms. (3) The energy was minimized and maintained close to the model protein structure by removing bad steric contacts [66]. For energy minimization, the OPLS 2005 force field was applied with an implicit solvation model [67].

### Molecular dynamics simulation studies

Combining molecular docking studies with Molecular dynamic simulations allows the validation of the docking results by confirming the structural stability and conformational flexibility of the ligand–protein complexes [68, 69]. The protein–ligand complexes are set to interact in a simulated environment for a specific time. Then, trajectories are computed for each protein–ligand complex, affording data about the molecular motions as a function of time [70]. The protein complexes of the five top hit compounds (CDI484583, ENA153723, 3lp2_LP9, ZINC000003986735, and Compound13509), and the crystal structure of *Streptococcus pneumoniae* Undecaprenyl pyrophosphate Synthase (UPPS) (PDB ID: 5KH5) [38] in complex with the pyrazole inhibitor **6TC** and the protein 5KH5 without the reference **6TC** underwent a 100 ns molecular dynamic simulations, thus enabling the study of the stability of these complexes.

Force fields are of great importance in biomolecular simulation as they calculate the potential energy of the particles of the complexes [71]. In this work, the Amber ff19SB force field [72] was used to generate topology and build a simulation box, while the general AMBER force field (GAFF) [73] was used for the ligands. A dodecahedral box of 12 Å was constructed around the protein–ligand complexes, and the systems were solvated after applying the forcefields. Solvation is essential in simulations as it enables studying the internal motion of protein systems at varying temperatures. The systems were solvated with TIP3P water, and charges were neutralized by adding sodium and chloride ions at a molar concentration of 0.15 M, leading to a constrained orthorhombic periodic cell, hence avoiding the formation of artifacts and giving the system a total charge of zero to minimize polarization [74–76].

The systems underwent energy minimization to relax the water molecules and intramolecular steric clashes; this was achieved at a temperature of 298 K under 1 bar pressure. The systems were subsequently equilibrated for 5000 ps with an integration time step of 2 fs, and the intermediate results were saved at 100 ps time intervals. A positional restraint of 700 kJ/mol was imposed on all bond lengths involving hydrogen atoms. Equilibration ensures that the kinetic and potential energy pumped through the system is appropriately supplied across all degrees of freedom [77].

The last step of operating molecular dynamics simulations is running the dynamics (Production) through a precise thermodynamic ensemble. In the Production phase, the constraints on the protein are removed. The system is allowed to run dynamics and generate trajectories of the protein and ligand atoms according to certain equilibrium conditions, such as the NPT ensemble (N: number of particles, P: pressure, and T: temperature), also known as the canonical ensemble [78] which was used for all the simulations. Temperature was maintained at 298 K using the Langevin thermostat [79], with a collision frequency of = 1/ps. The system was coupled to a Monte Carlo barostat [80] at a reference pressure of 1 atm and a relaxation time of 2 ps to achieve pressure control. After all the environment parameters are stated, the setup is set to observe for 100 ns. The data will be gathered for interpretation after the completion of the production. The production is displayed at 100 ps intervals.

All simulations in this work were done using the GPU-accelerated version of OpenMM 7.6 [81] engine and the ‘Making it rain’ [82] cloud-based molecular simulations notebook environment. Overall, 100 ns of MD simulations were attained for each system. The trajectories generated during the MD simulations of the protein–ligand complexes were analyzed to calculate the RMSD, RMSF, and the radius of gyration using scripts included in AMBER.

### Binding-free energies of protein–ligand complexes

The binding free energies (ΔG) of the protein–ligand complexes signify their binding affinity and thermodynamic stability, which directly corresponds to a compound’s potency [83]. In this study, Poisson–Boltzman (MM-PBSA) and Generalized Born (MM-GBSA) based approaches were used to calculate the ΔG of all the conformations formed during the 100 ns simulation. In general, the free energy (G) of the ligand, or the protein, is computed according to the following equation [84]:

1 $$G = \Delta E_{bind} + \Delta E_{elec} + \Delta E_{vDW} + G_{pol} + G_{np} - TS$$

where the free energy (G) is the sum of binding energy ${( \Delta E}_{bind})$, electrostatic interactions (${\Delta E}_{elec}$), van der Waals interactions (${\Delta E}_{vDW}$), polar energy (${G}_{pol}$) and non-polar energy (${G}_{np}$). In MMPBSA, the polar energy is obtained by solving the Poisson–Boltzman equation [85], while in the case of MMGBSA, the Generalized-Born model is used [86]. T and S are the absolute temperature and the entropy, respectively, which were excluded from our calculations. The binding free energies (${\Delta G}_{bind}$) were calculated using the following equation [85]:

2 $$\Delta G_{bind} = \Delta G_{complex} - \Delta G_{receptor} - \Delta G_{ligand}$$

where ${\Delta G}_{complex}$, ${\Delta G}_{receptor}$ and ${\Delta G}_{ligand}$ represent the free energy of the complex, receptor, and ligand.

## Results and discussion

### Generation of the 3D QSAR pharmacophore model

**Fig. 4:**
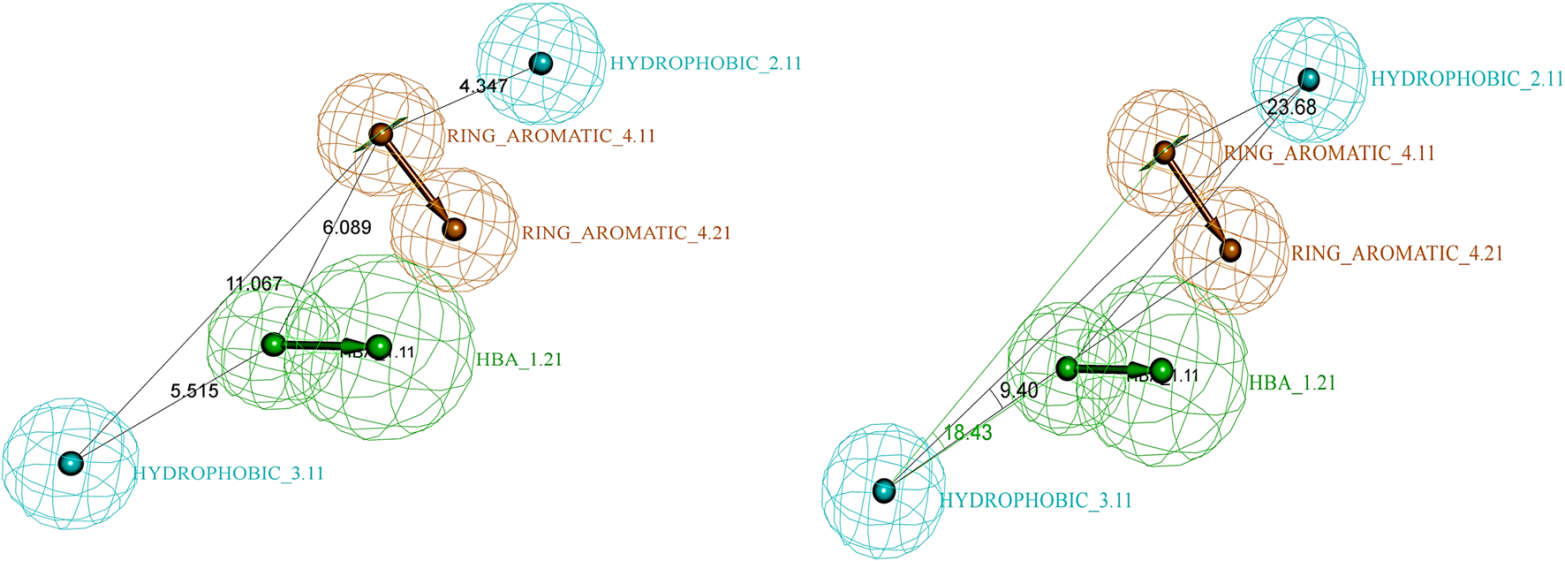
Spatial arrangement of the valid pharmacophore model with the distances and angles displayed

A training set of 25 structurally diverse compounds with their experimental IC_50_ values ranging from 0.04 to 35 μM [36] was used to generate a 3D QSAR pharmacophore model (Fig. 4). Utilizing the HYPOGEN algorithm, ten Hypotheses were created. The total cost, cost differences, RMSD, and correlation coefficient were the statistical parameters used to evaluate the ten generated pharmacophores (Table 1). The null cost for the generated Hypotheses is 332.267. The total costs for the ten generated Hypotheses ranged from 140 to 197.22. The total cost value of the best pharmacophore should be close to the fixed cost and distant from the null cost. Hypo 1 presented the highest null cost difference of 191.39, which means the pharmacophore model is more than 90% statistically significant. Besides the cost analysis, Hypo1 scored the highest correlation coefficient value of 0.86 and the lowest RMS value of 2.30504, attributing to a superior capacity for biological activity prediction. The selected pharmacophore model (Hypo 1) (Fig. 4) incorporates four chemical features: one hydrogen bond acceptor (HBA), two hydrophobic (HYD), and one ring aromatic (RA). The distances and angles between those features are shown in Table 2 and Fig. 4. The most valid pharmacophore, Hypo 1, was chosen for all subsequent screenings.

**Table 1:** Statistical parameters of top 10 generated pharmacophore models

Hypo No	Maximum fit	Total cost	Cost Distance	RMS	Correlation coefficient (r)	Features
1	7.6279	140.872	191.39	2.3050	0.8699	HBA HYD HYD RA
2	7.0756	148.201	184.07	2.4248	0.8549	HBA HYD HYD RA
3	7.2516	159.58	172.69	2.6073	0.8299	HBA HYD HYD HYD
4	7.1597	163.686	168.58	2.6688	0.8209	HBA HYD HYD HYD
5	8.0987	167.976	164.29	2.7243	0.8126	HBA HBA HYD HYD HYD
6	9.1462	183.64	148.63	2.9539	0.7750	HBA HBD HYD HYD HYD
7	6.0757	187.008	145.26	2.9559	0.7747	HBA HBD HYD HYD HYD
8	7.1269	190.955	141.31	3.0500	0.7577	HBA HBD HYD HYD
9	6.6619	192.426	139.84	3.0646	0.7550	HBD HYD HYD RA
10	6.9375	197.277	134.99	3.1088	0.746826	HBA HBA HYD HYD HYD

**Table 2:** The inter-features of the valid pharmacophore model: constraint distances and angles

Features	Constraint Distance (Å)
HYDROPHOBIC_3.11: HBA_1.21 HYDROPHOBIC_3.11: RING_AROMATIC_4.21 HYDROPHOBIC_3.11: HYDROPHOBIC_2.11 RING_AROMATIC_4.21: HYDROPHOBIC_2.11 RING_AROMATIC_4.21: HBA_1.21 HBA_1.21: HYDROPHOBIC_2.11	5.515 11.065 15.144 4.347 6.089 9.314

	Constraint Angles (°)
HYDROPHOBIC_3.11, HBA_1.21, RING_AROMATIC_4.21 RING_AROMATIC_4.21, HBA_1.21, HYDROPHOBIC_2.11 HYDROPHOBIC_2.11, RING_AROMATIC_4.21, HYDROPHOBIC_3.11	18.43 23.68 6.67

### Validation of the generated pharmacophore model

Three validation methods were applied to validate the chosen pharmacophore model Hypo 1: test set analysis, cost analysis, and Fischer’s randomization.

#### Test set analysis

Evaluating the pharmacophore models’ ability to predict biological activity was done via a test set of nine diverse UPPS inhibitors [36, 42] with varying IC_50_s (Fig. 3). The test set was mapped to the generated pharmacophore using the Ligand Pharmacophore Mapping tool, and estimated activities were calculated for each compound. The experimental and predicted activities of the training set and test set are stated in Tables 3 and 4. By comparing the estimated activity with the reported biological activity, we evaluated the accuracy of the pharmacophore Hypothesis’s ability to predict the test set’s activity. The test set scored a high correlation coefficient of 0.8177, indicating good prediction ability.

**Table 3:** Estimated and experimental activity of the training set

Name	Experimental activity	Estimated activity	Fit value
T1	0.04	0.0894	6.8886
T2	0.06	0.0482	7.1567
T3	0.07	2.2388	5.4900
T4	0.11	0.0868	6.9013
T5	0.11	0.1986	6.5419
T6	0.12	0.0703	6.9926
T7	0.14	0.1214	6.7557
T8	0.16	0.2673	6.413
T9	0.2	0.1177	6.7691
T10	0.2	0.2184	6.5008
T11	0.3	0.4343	6.2022
T12	0.5	0.4190	6.2178
T13	0.8	0.9777	5.8498
T14	1.1	0.5721	6.0825
T15	1.3	2.8485	5.3854
T16	1.8	1.0760	5.8082
T17	2	7.3483	4.9739
T18	2.1	1.7426	5.5988
T19	2.5	2.6886	5.4105
T20	3.5	2.3478	5.4694
T21	4.2	2.2259	5.4925
T22	8.5	3.3608	5.3136
T23	8.8	1.8356	5.5763
T24	24	12.455	4.7447
T25	35	16.954	4.6108

**Table 4:** Estimated and experimental activity of the test set

Name	Experimental activity	Estimated activity	Fit value
S1	58	1.52845	5.6558
S2	1.1	0.0262673	7.4206
S3	0.63	0.0250445	7.4413
S4	0.5	0.0446244	7.1905
S5	8.4	7.0014	4.9949
S6	11	1.71703	5.6053
S7	9.5	1.58512	5.6400
S8	9.2	6.60916	5.0199
S9	4.9	2.5735	5.4295

#### Cost analysis

The HypoGen algorithm in the Discovery Studio software was used to calculate three cost functions for validation. The fixed cost assumes a simple Hypothesis model that seamlessly fits all the dataset and library molecules; hence, it is the lowest cost [51]. The fixed cost value for the generated hypothesis is 74.40. The null cost, on the other hand, is equivalent to the highest possible error cost [52]. The null cost for the generated Hypotheses is 332.267. The total cost is calculated independently for each pharmacophore Hypothesis. It is the sum of weight, error, and fixed costs [44]. The total costs for the ten generated Hypotheses ranged from 140 to 197. Hypo 1 was found to have the greatest cost difference of 191.39 (Table 1), which means the pharmacophore model is statistically significant at more than 90%. Hypo1 also scored the highest correlation coefficient value of 0.8699 and the lowest RMS value of 2.3, both attributed to a superior capacity for biological activity prediction.

#### Fischer’s randomization

**Fig. 5:**
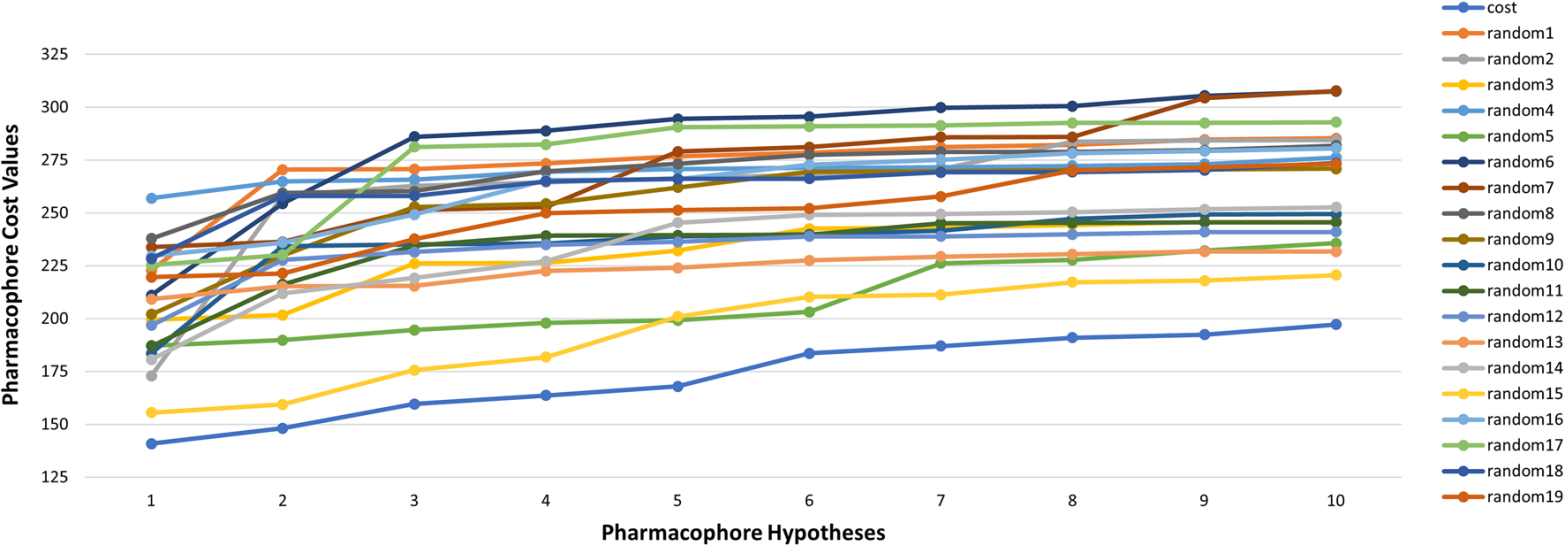
The difference in total cost values of hypotheses between a Hypo1 spreadsheet and 19 random spreadsheets

**Fig. 6:**
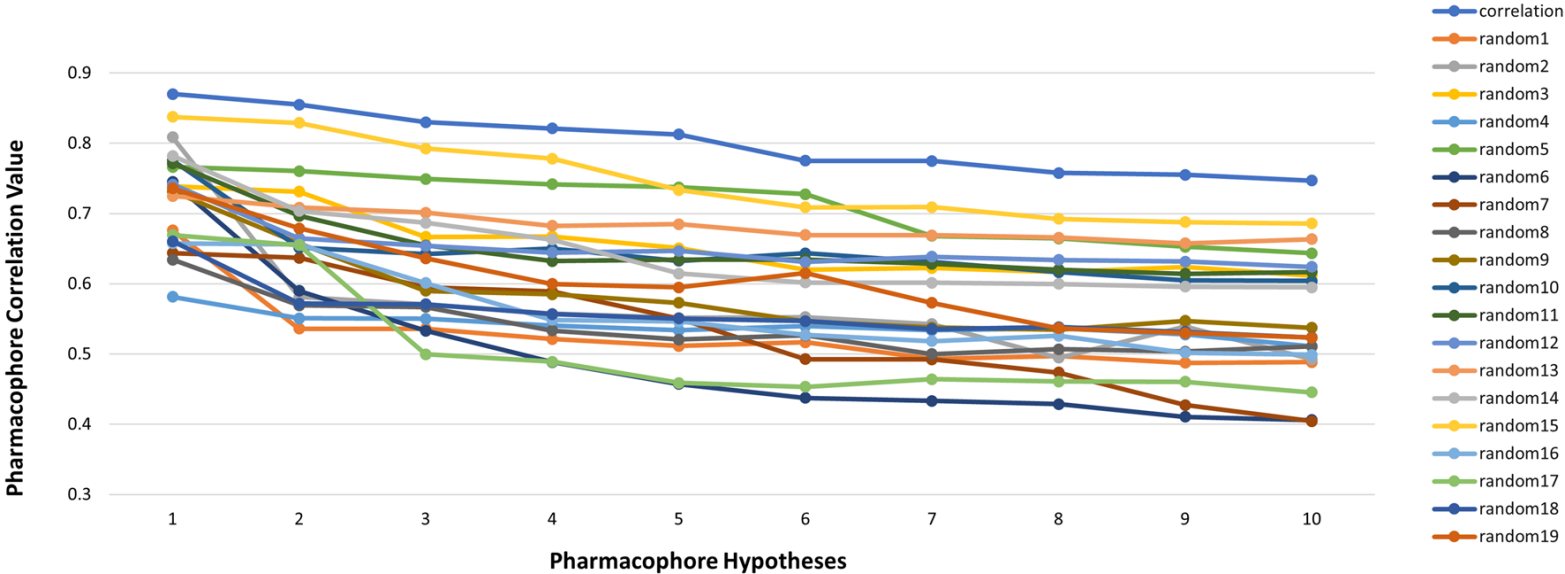
The difference in correlation values of hypotheses between a Hypo1 spreadsheet and 19 random spreadsheets

To obtain 95% confidence, 19 random spreadsheets (random Hypotheses) were created [78, 79]. Activity values were randomly assigned to the training set molecules using random spreadsheets, and Hypotheses were generated using the same features and parameters developed for Hypo1. Upon comparing the HypoGen pharmacophores and Fischer randomization, none of the randomly generated pharmacophores had greater statistical significance than Hypo1. Hypo1 displayed better total cost values (Fig. 5) and higher correlation values (Fig. 6) compared to the 19 random spreadsheets created by Fischer’s randomization. Fischer’s randomization method proves that Hypo1 is not a product of chance since it has greater significance than all random Hypotheses [55].

### Mapping of reference compound 6TC on the validated model

**Fig. 7:**
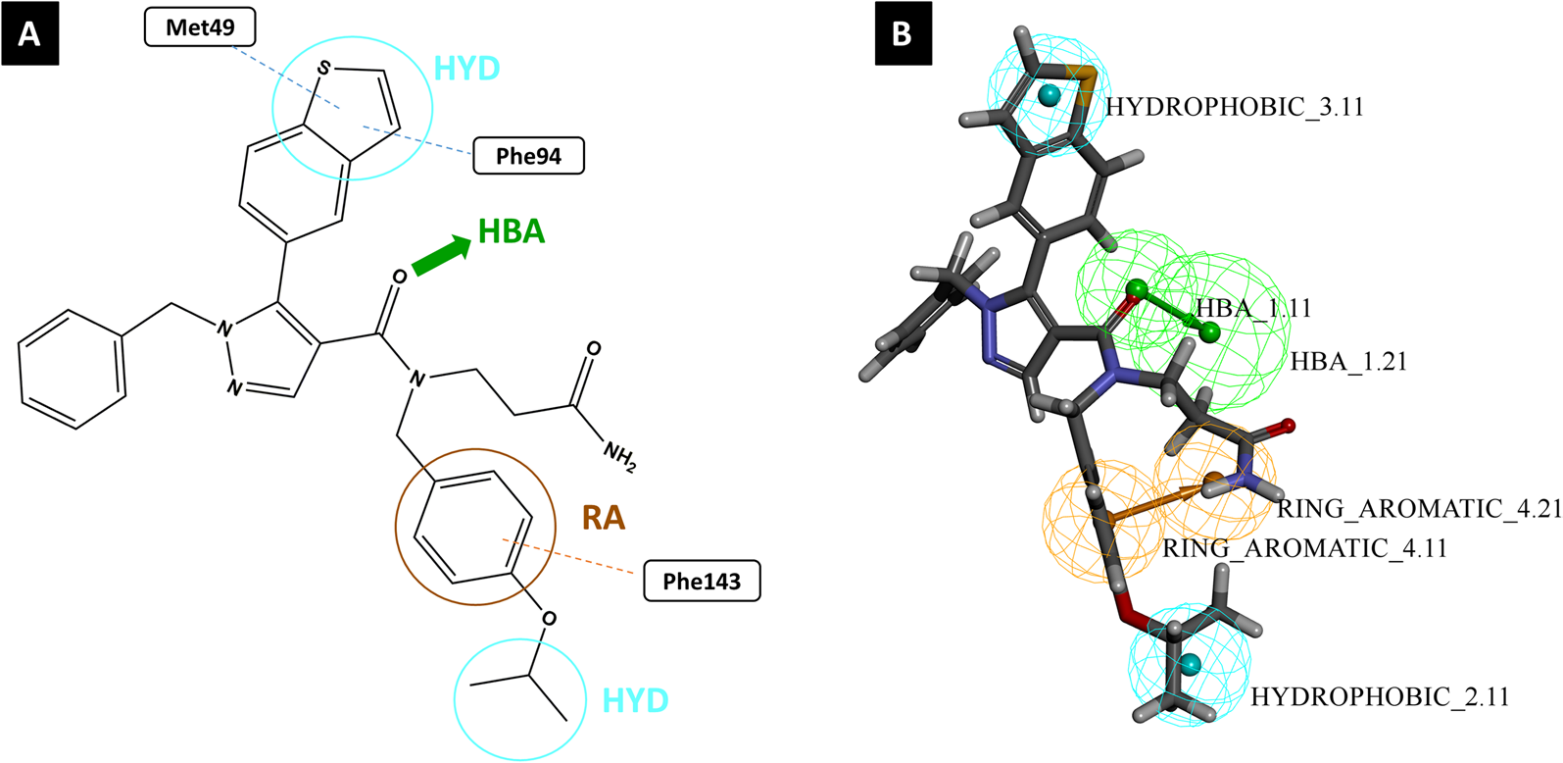
A. The 2D structure of the reference drug with reported interactions highlighted together with the chemical features. B. The mapping of the reference drug into HYPO1 with a fit value of 7.57 and an estimated IC_50_ of 0.018 µM

To further validate the chosen pharmacophore model, the reference ligand (**6TC**), co-crystalized in the target UPPS enzyme (PDB ID: 5KH5), was mapped via the Ligand Pharmacophore Mapping protocol in DS on the chosen pharmacophore model. The reference ligand successfully mapped into the pharmacophore’s four features (Fig. 7B) with a high fit value of 7.57. We also analyzed and compared how the reference fits into the model features with the interactions between the reference and the binding site of 5KH5 (Fig. 7A) [38]. In the reference ligand **6TC**, the benzothiophene moiety fits in a hydrophobic feature, consistent with the reported hydrophobic interactions between the benzothiophene moiety and amino acid residues Phe94 and Met49 [38]. The benzyl-isopropyl moiety fits in the ring aromatic feature of our pharmacophore model and is reported to have a pi-stacking interaction with the amino acid Phe143 [38]. This indicates that the docking and the pharmacophore model results consistently validate the computational process. **6TC** was used as a standard against which hits of the virtual screening were compared.

### Virtual screening

**Fig. 8:**
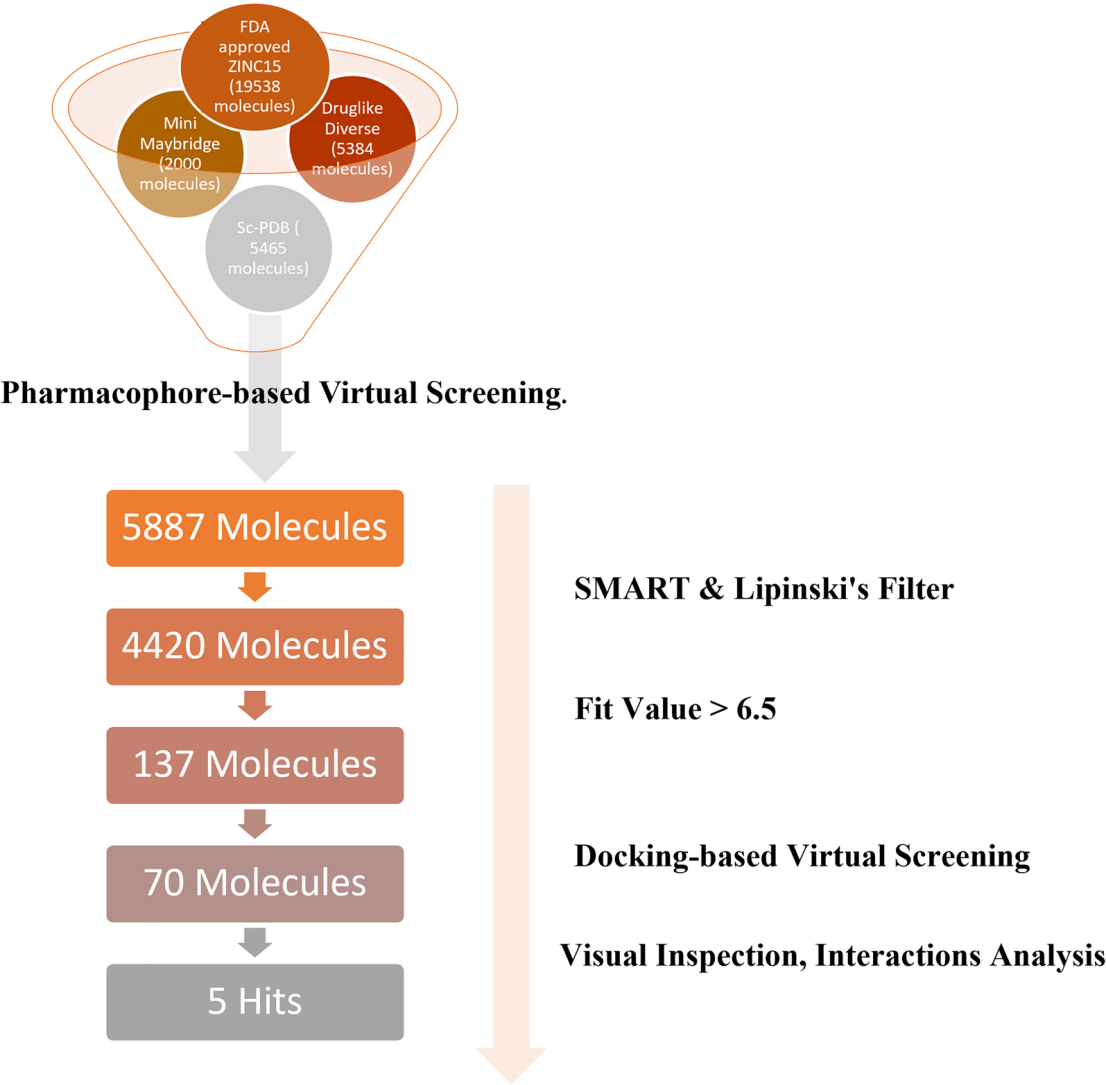
Schematic representation of the virtual screening process used to identify potential hits

The validated pharmacophore model was utilized to screen various databases, including FDA-approved molecules from the ZINC15 library [57], drug-like Diverse database, Mini Maybridge, and scPDB. 5887 of the 32387 screened molecules fitted into the validated pharmacophore model. The selected hits were subjected to Constraints like Lipinski’s filter and SMART filter, which were applied to ensure the drug-likeability of the selected hits. Both filters reduced the number of hits to 4420. Furthermore, Compounds with fit values close to the fit value of the reference were selected for docking-based virtual screening. The virtual screening process is represented in a schematic representation (Fig. 8).

### Molecular docking studies

#### Docking of the reference ligand *6TC*

Upon redocking the reference compound **6TC** into its binding site in the UPPS receptor (PDB ID: 5KH5) using the CDOCKER protocol in the DS, it confirmed the same orientation geometry mentioned in the literature. Furthermore, the generated top five docking poses were compared to the original crystallized reference ligand **6TC** by computing the Root Mean Square Deviation (RMSD). The RMSD value was equal to 0.92 Å, successively validating the docking protocol applied. This ensures the validity of using the docking results of **6TC** as a reference against which all the hits generated by the pharmacophore-based virtual screening are compared.

**Fig. 9:**
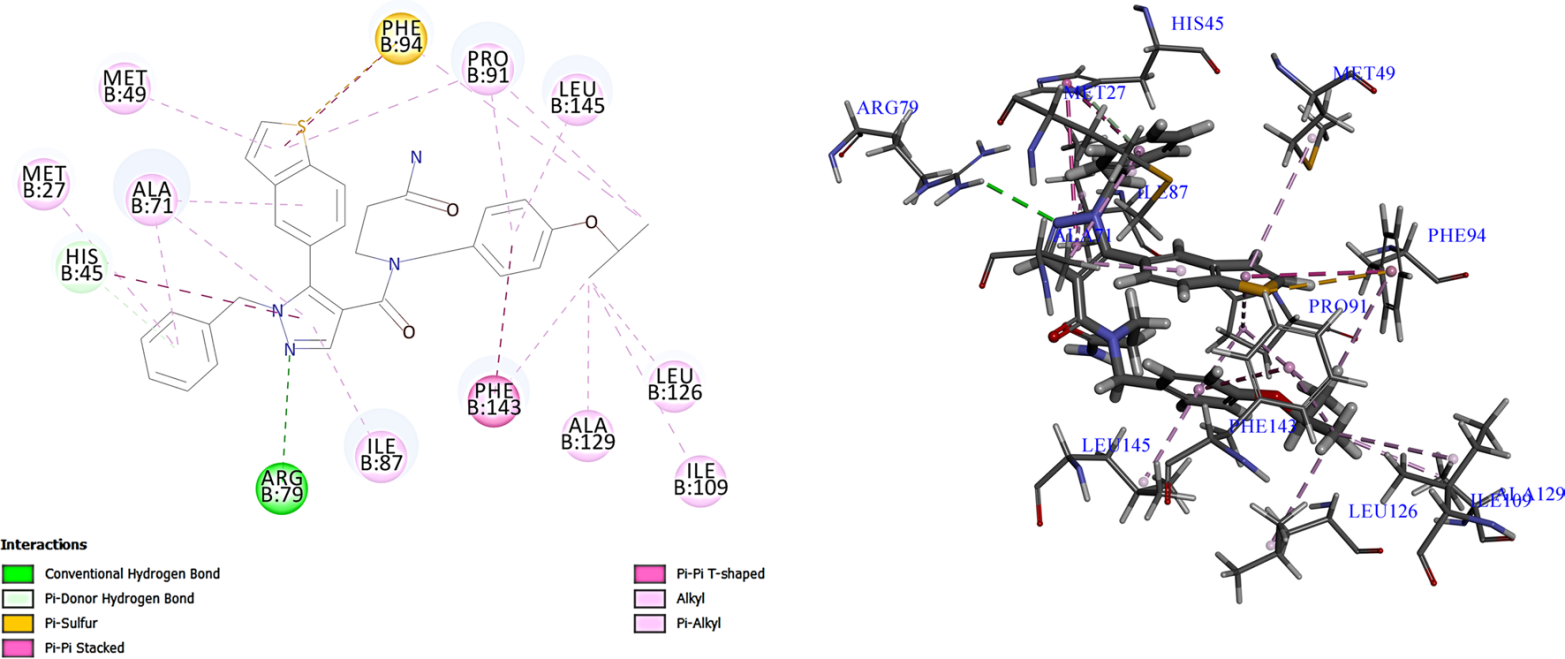
The 2D and 3D representation of the docking of the reference ligand (6TC) inside the binding site of UPPS (PDB ID: 5KH5)

The inhibitor **6TC** scored a -CDOCKER energy of 21.17 kcal/mol and forms two hydrogen bonds, one direct hydrogen bond between the pyrazole moiety and **Arg79** (Fig. 9). The other indirect pi donor hydrogen bond is between the pyrazole moiety and **His45.** The inhibitor forms mostly hydrophobic interactions similar to the natural substrate FPP [38]. The isopropoxy benzyl moiety fits into a hydrophobic pocket and forms nine hydrophobic interactions with the amino acids (Phe143, Ala129, Ile109, Leu126, Leu 145, Pro91, Phe94), while the Benzo-thiophene moiety fits into a hydrophobic pocket and forms three hydrophobic interactions with the amino acids (Pro91, Met49, Ala71) and the pyrazole moiety formed three hydrophobic interactions with the amino acids (Ala71, Ile78, His45). In addition, the benzyl moiety forms two hydrophobic interactions with the amino acids (Ala71, Met27). Lastly, the Benzo-thiophene moiety forms one electrostatic interaction with the amino acid residue Phe94. All interactions and docking results are presented in (Fig. 9) (Tables 5 and 6).

**Table 5:**
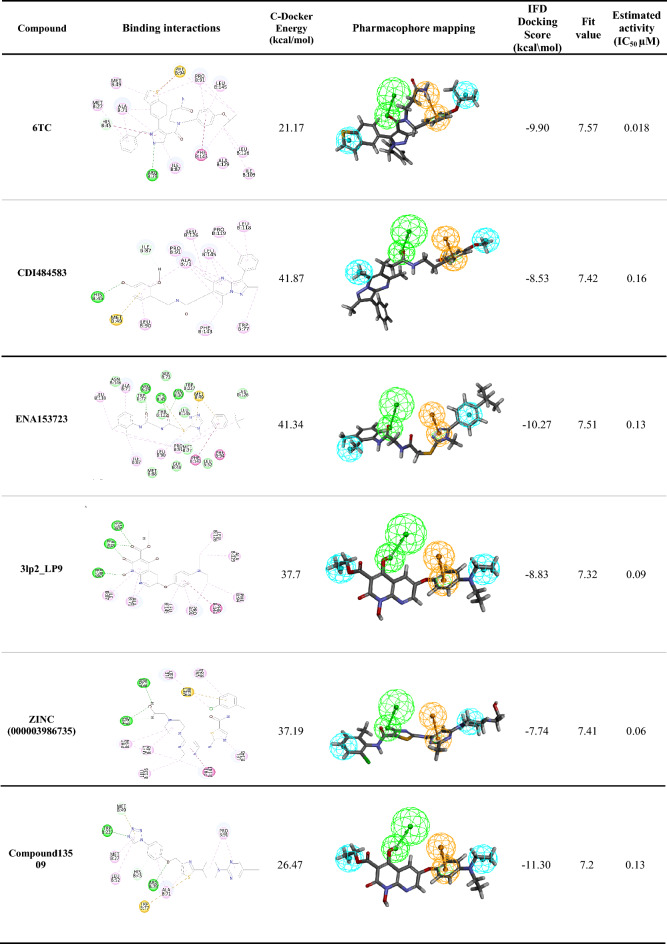
Comparison between 6TC and the five top hits selected from virtual screening with their docking scores, IFD docking scores, mapping pharmacophore features, pharmacophore fit values, and estimated activities

The hydrogen bond acceptor (HBA) is colored green, the ring aromatic (RA) is orange, the positive ionizable feature (PI) is red, and the hydrophobic area (HYD) is cyan

**Table 6:** Docking results of 6TC and the selected hits from the docking-based virtual screening stating the hydrogen bonds and hydrophobic interactions

Compound	Hydrogen bonds	H-bond length A°	Hydrophobic interactions
**6TC (Ref)**	**Arg79—N of pyrazole** **His45—phenyl moiety**	3.05 3.19	$$\left. \begin{gathered} {\mathbf{Phe143}} \hfill \\ {\mathbf{Ala129}} \hfill \\ {\mathbf{Ile109}} \hfill \\ {\mathbf{Leu126}} \hfill \\ {\mathbf{Leu}} \, {\mathbf{145}} \hfill \\ {\mathbf{Pro91}} \hfill \\ {\mathbf{Phe94}} \hfill \\ \end{gathered} \right\}{\text{Isopropoxybenzyl moiety}}$$ $$\left. \begin{gathered} {\mathbf{Pro91}} \hfill \\ {\mathbf{Met49}} \hfill \\ {\mathbf{Ala71}} \hfill \\ \end{gathered} \right\}{\text{Benzo}} - {\text{thiophene moiety}}$$ $$\left. \begin{gathered} {\mathbf{Ala71}} \hfill \\ {\mathbf{Ile78}} \hfill \\ {\mathbf{His45}} \hfill \\ \end{gathered} \right\}{\text{Pyrazole moiety}}$$ $$\left. \begin{gathered} {\mathbf{Ala71}} \hfill \\ {\mathbf{Met27}} \hfill \\ \end{gathered} \right\}{\text{Benzyl moiety}}$$
**CDI484583**	**His45—O of dimethoxybenzyl** Ile87—H of dimethoxybenzyl	2.68 2.35	$$\left. \begin{gathered} {\mathbf{Phe143}} \hfill \\ {\mathbf{Leu126}} \hfill \\ {\mathbf{Leu145}} \hfill \\ {\mathbf{Ala71}} \hfill \\ {\mathbf{Pro91}} \hfill \\ {\text{Trp77}} \hfill \\ \end{gathered} \right\}{\text{Trimethyl pyrazolopyrimidine moiety}}$$ $$\left. \begin{gathered} {\text{Leu118}} \hfill \\ {\text{Pro119}} \hfill \\ \end{gathered} \right\}{\text{Phenyl moiety}}$$ $$\left. \begin{gathered} {\mathbf{Ala71}} \hfill \\ {\text{Leu9}}0 \hfill \\ \end{gathered} \right\}{\text{Dimethoxybenzyl moiety}}$$
**ENA153723**	**Arg79—O of acetamide** **His45—O of acetamide** Asn30—O of acetamide	2.6 2.14 2.37	$$\left. \begin{gathered} {\mathbf{Phe143}} \hfill \\ {\mathbf{Pro91}} \hfill \\ {\mathbf{Phe94}} \hfill \\ {\text{Met94}} \hfill \\ \end{gathered} \right\}{\text{Isobutyl phenyl moiety}}$$ $$\left. \begin{gathered} {\text{Met94}} \hfill \\ {\text{Leu9}}0 \hfill \\ \end{gathered} \right\}{\text{Triazole moiety}}$$ $$\left. \begin{gathered} {\mathbf{Pro91}} \hfill \\ {\mathbf{Ala71}} \hfill \\ {\text{Ile87}} \hfill \\ {\text{Leu118}} \hfill \\ \end{gathered} \right\}{\text{Dimethyl phenyl moiety}}$$
**3lp2_LP9**	**Arg79—O of hydroxy** **His45—O of carboxylate** **His45—O of carbonyl** Asn30—O of carboxylate	2.21 2.05 2.4 2.47	$$\left. \begin{gathered} {\mathbf{Phe143}} \hfill \\ {\mathbf{Ala129}} \hfill \\ {\mathbf{Leu126}} \hfill \\ {\mathbf{Leu145}} \hfill \\ {\mathbf{Pro91}} \hfill \\ {\mathbf{Phe94}} \hfill \\ \end{gathered} \right\}{\text{Diethyl amino phenoxy moiety}}$$ $$\left. \begin{gathered} {\mathbf{Ala71}} \hfill \\ {\text{Il87}} \hfill \\ \end{gathered} \right\}{\text{Naphthyridine moiety}}$$
**ZINC000003986735**	**His45—O of hydroxy**	2.6	$$\left. \begin{gathered} {\mathbf{Phe143}} \hfill \\ {\mathbf{Leu126}} \hfill \\ {\mathbf{Pro91}} \hfill \\ {\mathbf{Ile109}} \hfill \\ \end{gathered} \right\}{\text{Methyl pyrimidine moiety}}$$ $$\left. \begin{gathered} {\mathbf{Pro91}} \hfill \\ {\mathbf{Met49}} \hfill \\ {\mathbf{Phe94}} \hfill \\ \end{gathered} \right\}{\text{Piperazine moiety}}$$ $$\left. \begin{gathered} {\mathbf{Pro91}} \hfill \\ {\mathbf{Leu145}} \hfill \\ \end{gathered} \right\}{\text{Thiazole moiety}}$$ ${\text{Trp77}}$—Chloro substitution on methyl phenyl moiety
**Compound13509**	**Arg79—O of phenoxy moiety** **His45—Phenoxy moiety** Met49—Triazole moiety Trp77—N of triazole moiety	2.2 2.8 2.49 2.24	${\mathbf{Ala71}}$**—**Phenoxy moiety ${\mathbf{Ala71}}$—Thiazole moiety **Pro91—**Ethyl pyrimidine moiety **Pro91—**Piperidine moiety $$\left. \begin{gathered} {\text{Met27}} \hfill \\ {\text{Leu52}} \hfill \\ \end{gathered} \right\}{\text{Triazole moiety}}$$

#### Docking of the virtual screening hits

Following the pharmacophore-based virtual screening of various databases and further refining using SMARTS and Lipinski’s filters, compounds with fit values close to the reference compound **6TC** (> 6.5) were selected for docking-based virtual screening. The hits were docked onto the target UPPS protein (PDB ID: 5KH5) [38] using the CDOCKER protocol in DS. Seventy compounds were found to have higher docking energies than the reference and were filtered according to careful visual inspection and comparison to the interactions made by the reference. The reference **6TC** forms two hydrogen bonds, one direct hydrogen bond between the pyrazole moiety and **Arg79** and the other an indirect pi donor hydrogen bond between the pyrazole moiety and **His45.** It also fits into several hydrophobic pockets, forming several hydrophobic bonds, as illustrated in Tables 5 and 6. The interactions of the 70 compounds were compared to those of the reference. Five hits were found to have very similar interactions and higher docking energies, indicating potential UPPS inhibition and antibacterial activity (Tables 5 and 6).

##### Docking Studies of CDI484583

CDI484583, also known as ZINC9609856, scored a -CDOCKER energy of 41.87. The hit compound formed two hydrogen bonds: the O of the dimethoxy benzyl formed one direct hydrogen bond with His45, and the H of dimethoxy benzyl formed one indirect hydrogen bond with Ile87. The phenyl moiety and the trimethyl pyrazolopyrimidine moiety fit into a hydrophobic pocket and form a total of 13 hydrophobic interactions with the amino acids (Phe143, Leu126, Leu145, Ala71, Pro91, Trp77, Leu118, Pro119). The Dimethoxybenzyl moiety forms two hydrophobic interactions with the amino acids (Ala71, Leu90). The hit compound also makes an electrostatic interaction between the phenyl moiety and Met45. When mapped to the generated valid pharmacophore, the compound scored a high fit value of 7.42 and had similar interactions to the reference compound and higher docking energies (Tables 5, 6).

##### Docking studies of ENA153723

ENA153723 scored a -CDOCKER energy of 41.34. The hit compound formed three direct hydrogen bonds: the two Oxygens of acetamide moiety with the His45, Arg79, and Asn30. The isobutyl phenyl moiety fits into a hydrophobic pocket and forms a total of four hydrophobic interactions with the amino acids (Phe143, Pro91, Phe94, Met94), the dimethyl phenyl moiety fits into a hydrophobic pocket and forms five hydrophobic interactions with the amino acids (Pro91, Ala71, Ile87, Leu118), and the triazole moiety forms two hydrophobic interactions with the amino acid residues Met94 and Leu90. The hit compound also makes three electrostatic interactions, one between the sulphur of acetamide moiety and His45 and two between the isobutyl phenyl moiety and triazole moiety, and the amino acid Met49. When mapped to the generated valid pharmacophore, The compound scored a high fit value of 7.51 in addition to having very similar interactions to the reference compound and higher docking energies (Tables 5, 6).

##### Docking studies of 3lp2_LP9

3lp2_LP9 scored a -CDOCKER energy of 37. The hit compound formed four direct hydrogen bonds between the oxygen of hydroxy, oxygen of carboxylate, and oxygen of the carbonyl group and the amino acids (His45, Arg79, Asn30). The diethyl amino phenoxy moiety fits into a hydrophobic pocket. It forms a total of seven hydrophobic interactions with the amino acids (Phe143, Ala129, Leu126, Leu145, Pro91, Phe94), and the naphthyridine moiety forms two hydrophobic interactions with the amino acid residues Ala71 and Il87. When mapped to generate a valid pharmacophore, The compound scored a high fit value of 7.32 in addition to having very similar interactions to the reference compound and higher docking energies (Tables 5, 6).

##### Docking studies of ZINC000003986735

ZINC000003986735 scored a -CDOCKER energy of 37.19. The hit compound formed two direct hydrogen bonds between the oxygen of the hydroxy group and the amino acid His45. The Piperazine moiety and the Methyl pyrimidine moiety fit into a hydrophobic pocket and form a total of nine hydrophobic interactions with the amino acids (Phe143, Leu126, Pro91, Ile109, Met49, Phe94), and the Thiazole moiety forms two hydrophobic interactions with the amino acid residues Pro91 and Leu145. In addition, the chloride substitution on methyl phenyl moiety forms a hydrophobic interaction with Trp77. When mapped to the generated a valid pharmacophore, The compound scored a high fit value of 7.41 in addition to having very similar interactions to the reference compound and higher docking energies (Tables 5, 6).

##### Docking studies of compound13509

Compound 13,509 scored a -CDOCKER energy of 26.47. The hit compound formed three direct hydrogen bonds: two direct hydrogen bonds between the oxygen of phenoxy moiety, N of triazole and amino acids Arg79 and Trp77, and one indirect hydrogen bond between the phenoxy moiety and the amino acid His45. The thiazole and the phenoxy moieties form hydrophobic interactions with the amino acid residue Ala71, and the ethylpyrimidine and Piperidine moieties form hydrophobic interactions with the amino acid residue Pro91. In addition, the triazole moiety forms two hydrophobic interactions with amino acid residues Met27 and Leu52. The hit compound also makes one electrostatic interaction between the nitrogen of thiazole moiety and the amino acid Trp77. When mapped to the generated valid pharmacophore, the compound scored a high fit value of 7.2 and had reasonably similar interactions to the reference compound and higher docking energies (Tables 5, 6).

### Induced fit docking

Molecular docking offers a good start to evaluate the stability of the predicted interactions involved in a ligand’s binding [67]. IFD is a combined convention of molecular docking and dynamics, it helps investigate the active site’s dynamic nature during ligand binding [66]. In this study, the selected top five hits and the reference compound 6TC were docked in the active site of the UPPS receptor (PDB ID: 5KH5). The IFD scores for compounds 6TC, CDI484583, ENA153723, 3lp2_LP9, ZINC000003986735, and Compound13509 are shown in Table 5. The reference compound (6TC) had an IFD score of − 9.90 kcal/mol, while the top five hits had IFD scores ranging from − 11.30 to 7.74 kcal/mol. IFD confirmed that the selected top five compounds were well bound to the active site of UPPS with good IFD scores and displayed molecular interactions similar to the reference compound 6TC (Additional file 1: Table S1).

### Molecular dynamics simulation studies

To further validate the docking results of the screened molecules in the binding site of UPPS(PDB ID: 5KH5) [38], we simulated the protein complexes of the five top hit compounds (CDI484583, ENA153723, 3lp2_LP9, ZINC000003986735, and Compound13509) as well as, 5KH5 alone and 5KH5 in complex with the reference compound using Amber ff19SB force field [72] and the general AMBER force field (GAFF) [73] through the GPU-accelerated version of OpenMM 7.6 [81] engine and the ‘Making it rain’ [82] cloud-based molecular simulations notebook environment. A 100 ns of MD simulation was attained for each system, and A 1000-frame trajectory was generated by combining a production simulation duration with a production save results interval of 100 ps. The trajectories generated during the MD simulations of the protein–ligand complexes were analyzed to calculate the RMSD, radius of gyration, and RMSF values using scripts included in AMBER (Table 7).

**Table 7:** The average values of Root Mean Square Deviation (RMSD), the radius of gyration (ROG), and Root Mean Square fluctuations (RMSF) for the protein–ligand complexes during a 100 ns MD run

Complex	RMSD	ROG	RMSF
5KH5-6TC	2.64	18.04	1.48
5KH5-Top hit 1	1.70	17.98	1.11
5KH5-Top hit 2	1.90	17.86	1.21
5KH5-Top hit 3	1.97	17.99	1.16
5KH5-Top hit 4	1.86	17.84	1.19
5KH5-Top hit 5	2.15	18.09	1.23

**Fig. 10:**
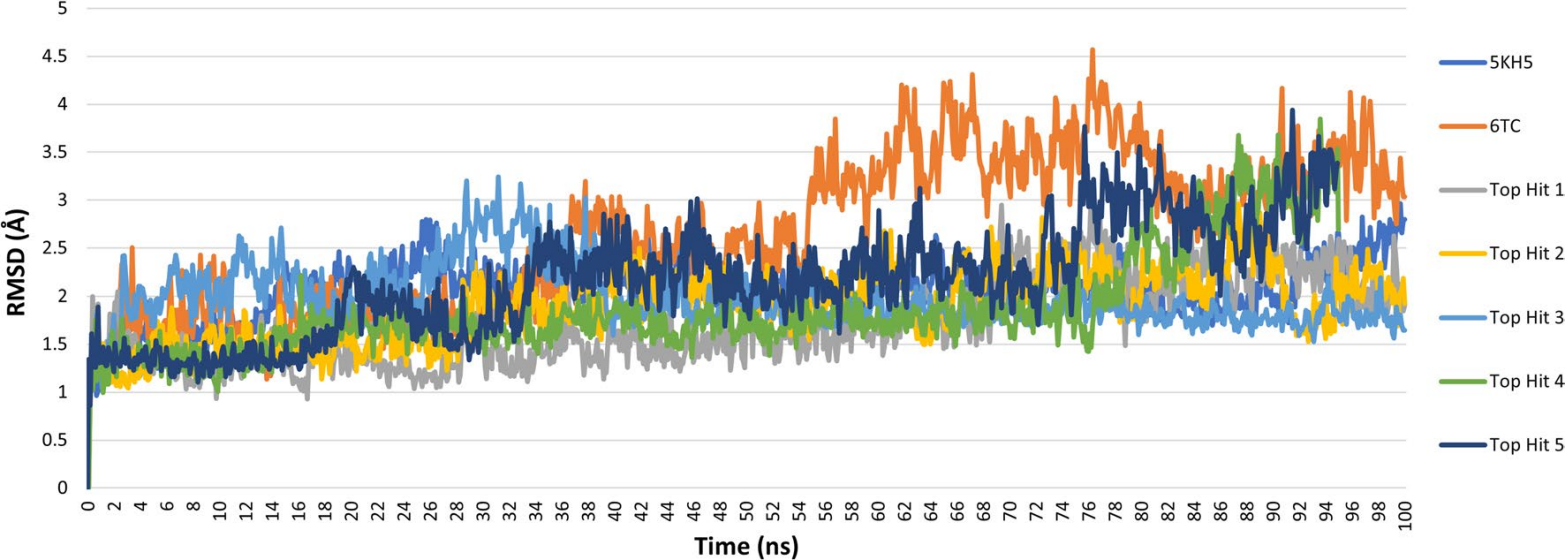
Root Mean Square Deviations (RMSD) of protein 5KH5 alone, the docked reference ( 6TC) and the selected top five hits

Root Mean Square Deviation (RMSD) assesses the difference in the backbone of a protein complex from its initial structural conformation to its final position. RMSD represents the extent of structural changes in the protein compared to the reference structure during the simulation run, providing a reliable measure of the stability of docking complexes [87–89]. The analysis of the RMSD for the top hit-protein complexes showed that they were largely stable throughout the simulation run with minor fluctuations (Fig. 10). The reference 6TC-protein complex had an average RMSD of 2.64 Å. The average RMSD of the top five hits ranged from 1.7 to 2.15 Å (Table 7), thus indicating that the top hits may have a better level of stability than the reference 6TC, as lower RMSD values indicate more stable systems [89, 90]. Moreover, top hits one, two, and three (ENA153723, 3lp2_LP9, and ZINC000003986735) showed the highest stability during the simulation run with an average RMSD value of 1.7, 1.9, and 1.97 Å, respectively and RMSD fluctuations of 0.19, 0.14 and 0.1 Å. Overall, the RMSD analysis suggests the systems’ stability and confirms the docking results’ credibility.

**Fig. 11:**
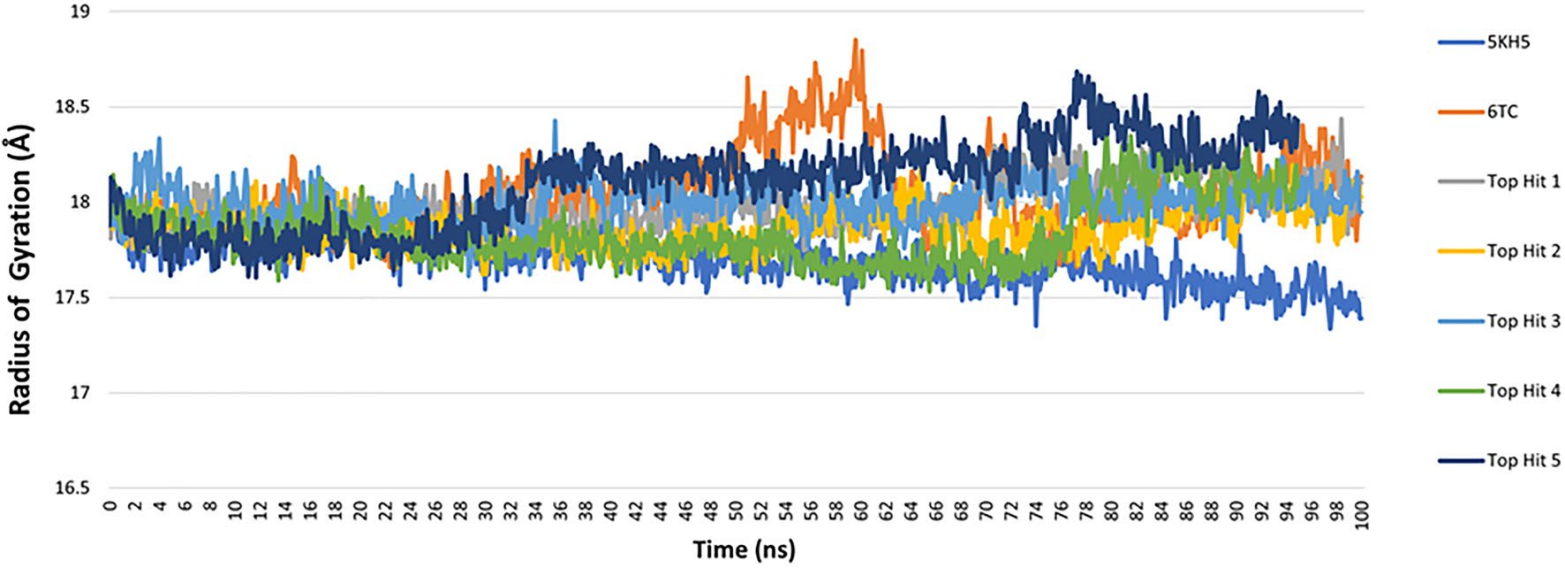
Radius of Gyration of protein 5KH5 alone, the docked reference 6TC and the selected top five hits

The radius of gyration (RG) is a measure of protein compactness and the stability of conformations [91]. Upon investigation of RG for the protein alone and the protein in complex with the reference 6TC and the top five hits, we concluded a tightly packed, stable protein folding while complexed with the ligands during the 100 ns long molecular dynamic run. The average radius of gyration for the reference **6TC** was equal to 18.04 Å, while the five top hits ranged from 17.86 to 18.09 Å (Table 7). According to these results, the hits are highly compact, as shown in Fig. 11.

**Fig. 12:**
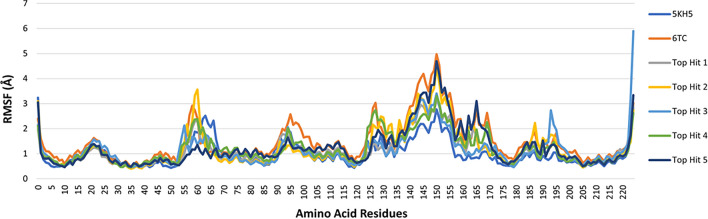
Root mean square deviation (RMSF) of protein 5KH5 alone, the docked reference 6TC and the selected top five hits

Root mean square fluctuation (RMSF) values indicate the extent of motion that amino acid residues in a protein experience, with higher values indicating greater mobility [85]. In regions with high RMSF values, the residues can move around more freely, allowing more flexibility. In regions with low RMSF values, the residues are more restricted, leading to rigidity [78, 92]. By investigating the RMSF values, we can assess the flexibility of residue side chains and backbone and, thus, the flexibility of the overall molecular dynamic simulation [93]. As shown in Fig. 12, all the protein–ligand complexes displayed similar flexibilities. The average RMSF value for the reference (**6TC**) complexed with the protein 5KH5 was equal to 1.48 Å, and the top five hits average RMSF values ranged from 1.11 to 1.23 Å (Table 7). Additional RMSF analysis showed that residues 136–156 of 5KH5 displayed high fluctuations in all systems. Residues Arg79, His45, Asn30, Ile87, Met49, and Try77 of 5KH5, which all mainly form ligand–protein hydrogen bonds, displayed low fluctuations in all systems.

The binding site residue interactions of the protein–ligand complexes were observed for the best energy conformations obtained from the MD run (Additional file 1: Table S2). Most of the protein residue interactions with the inhibitors are defined as hydrophobic. The reference ligand (6TC) and top hit 1 (CDI484583) formed hydrophobic and van der Waals interactions and no hydrogen bonds. Top hit two (ENA153723) formed two conventional hydrogen bond interactions with Asn14 and Hie29, while Top hit three (3lp2_LP9) formed one conventional hydrogen bond interaction with the amino acid residue Glu62. Top hits four and five (ZINC000003986735 and Compound13509) formed one conventional hydrogen bond interaction with amino acid residue Asn14.

### Binding free energies of protein–ligand complexes

The binding free energies for the interactions of the top five hits and the reference (6TC) with the UPPS receptor 5KH5 (PDB ID: 5KH5) [38] were computed using the Poisson–Boltzman (MM-PBSA) and Generalized Born (MM-GBSA) methods [84]. The results are shown in Table 8. The contribution of van der Waals (E_vdW_), electrostatic (E_elec_), polar solvation (E_surf_), non-polar solvation (E_npolar_), Generalized Born solvent (E_EGB_), and Poisson Boltzmann solvent (E_EPB)_ energies to the total binding free energies are also tabulated. Lower binding affinity implies strong interactions and better stabilities, revealing the compound’s potency [94]. According to MM-GBSA calculations, ΔG for the reference ligand 6TC was (− 47.125 kcal/mol). The binding free energies of the top five hits complexed with the UPPS protein 5KH5 ranged from − 52.240 to − 42.656 kcal/mol, of which the top hit four (− 52.240 ± 2.928 kcal/mol) had the lowest binding free energy. Top hits two, four and five displayed lower binding free energies than the reference 6TC, indicating better stabilities. As per MM-PBSA calculations, ΔG for the reference ligand 6TC was (− 0.973 kcal/mol). The binding free energies of the top five hits complexed with the UPPS protein 5KH5 ranged from − 3.081 ± 1.789 to 2.020 kcal/mol, of which the top hit three (− 1.702 ± 1.379) and the top hit five (− 3.081 ± 1.789 kcal/mol) had lower binding free energies than the reference 6TC indicating better stabilities.

**Table 8:** The average binding free energy (kcal/mol) of protein–ligand complexes during the 100 ns MD run using MM-GBSA and MM-PBSA methods

Complex	ΔE_vdW_	ΔE_elec_	ΔE_EGB_	ΔE_ESURF_	ΔG_gas_	ΔG_solv_	ΔG_bind_
**MM-GBSA**							
5KH5-6TC	− 67.364 ± 0.000	− 5.128 ± 0.000	33.456 ± 0.000	− 8.0885 ± 0.000	− 72.493 ± 0.000	25.367 ± 0.000	− 47.125 ± 0.000
5KH5-Top hit 1	− 58.883 ± 0.000	− 3.782 ± 0.000	26.950 ± 0.000	− 6.940 ± 0.000	− 62.666 ± 0.000	20.010 ± 0.000	− 42.656 ± 0.000
5KH5-Top hit 2	− 65.679 ± 0.937	− 17.530 ± 3.473	41.416 ± 2.894	− 7.724 ± 0.081	− 83.210 ± 3.420	33.692 ± 2.888	− 49.518 ± 1.013
5KH5-Top hit 3	− 57.009 ± 1.301	− 33.640 ± 2.566	53.189 ± 1.918	− 7.373 ± 0.098	− 90.649 ± 2.521	45.815 ± 1.925	− 44.833 ± 0.932
5KH5-Top hit 4	− 63.784 ± 2.687	− 19.368 ± 2.557	38.279 ± 2.054	− 7.368 ± 0.206	− 83.152 ± 4.578	30.911 ± 1.898	− 52.240 ± 2.928
5KH5-Top hit 5	− 61.684 ± 0.933	− 2.815 ± 0.382	23.045 ± 0.574	− 6.949 ± 0.567	− 64.500 ± 1.064	16.095 ± 0.573	− 48.404 ± 1.195

Complex	ΔE_vdW_	ΔE_elec_	ΔE_EPB_	ΔE_ENPOLAR_	ΔG_gas_	ΔG_solv_	ΔG_bind_
**MM-PBSA**							
5KH5-6TC	− 67.364 ± 0.000	− 5.128 ± 0.000	35.881 ± 0.000	− 46.028 ± 0.000	− 72.493 ± 0.000	71.519 ± 0.000	− 0.973 ± 0.000
5KH5-Top hit 1	− 58.883 ± 0.000	− 3.782 ± 0.000	32.393 ± 0.000	− 40.349 ± 0.000	− 62.666 ± 0.000	64.686 ± 0.000	2.020 ± 0.000
5KH5-Top hit 2	− 65.679 ± 0.937	− 17.530 ± 3.473	50.633 ± 2.421	− 43.790 ± 0.260	− 83.210 ± 3.420	83.267 ± 2.535	0.057 ± 2.058
5KH5-Top hit 3	− 57.009 ± 1.301	− 33.640 ± 2.566	61.030 ± 2.665	− 39.322 ± 0.477	− 90.649 ± 2.521	88.946 ± 2.806	− 1.702 ± 1.379
5KH5-Top hit 4	− 63.784 ± 2.687	− 19.368 ± 2.557	53.131 ± 3.455	− 41.448 ± 0.972	− 83.152 ± 4.578	85.068 ± 4.289	1.915 ± 1.265
5KH5-Top hit 5	− 61.684 ± 0.933	− 2.815 ± 0.382	30.604 ± 1.113	− 39.894 ± 0.262	− 64.500 ± 1.064	61.418 ± 1.288	− 3.081 ± 1.789

## Conclusion

This study generated a valid predictive pharmacophore model via the 3D QSAR pharmacophore generation protocol by utilizing a set of 34 UPPS inhibitors with known activity. The pharmacophore model (Hypo1) was validated by having the highest cost difference (191.39), the highest correlation coefficient (0.86), and the lowest total cost value (140.87). The model consists of four features: one hydrogen bond acceptor, two hydrophobics, and one ring aromatic. The Hypo1 model was cross-validated by test set predictions, cost analysis, and Fischer’s randomization, all of which confirmed the model’s high predictive power. The model was used to screen 32387 molecules from various databases. Five thousand eight hundred eighty-seven molecules fit into the validated pharmacophore model and were filtered by Lipinski’s and SMART filters to ensure the drug-likeability of the selected hits. Furthermore, compounds with fit values close to the fit value of the reference (> 6.5) were chosen for docking-based virtual screening. Compounds were docked using the CDOCKER protocol into the target UPPS binding site, and 70 hits had higher docking affinities than the reference **6TC**. After extensive docking analysis and visual inspection, five top hits were selected based on docking affinities, fit values, and key residue interactions. The top five hits are CDI484583, ENA153723, 3LP2_LP9, ZINC000003986735, and Compound13509. IFD confirmed that the selected top five compounds were well bound to the active site of UPPS with good IFD scores and displayed molecular interactions similar to the reference compound 6TC. The top five hits were subjected to MD simulations, which validated the stability of the binding mode, yielding five promising putative UPPS inhibitors. In vitro and in vivo biological testing would be valuable for further evaluating these inhibitors (Additional file 2).

### Supplementary Information

**Additional file 1: Table S1.** Induced fit docking interactions for the reference ligand (6TC) and the selected top five hits. **Table S2.** Interactions between the examined ligands (6TC and the selected top five hits) and the UPPS protein during the simulation run.

**Additional file 2.** Data sheets of the graphs presented in Figures 5, 6, 10, 11 & 12.

## Data Availability

This published article contains all the data produced or examined during this study.
