# Supplementary material for: Lead generation of UPPS inhibitors targeting MRSA: Using 3D-QSAR pharmacophore modeling, virtual screening, molecular docking, and molecular dynamic simulations
Source: BMC Chem. 2024 Jan 20;18(1):14. doi: 10.1186/s13065-023-01110-1 (PMC10800075; doi:10.1186/s13065-023-01110-1)
Supplement: Supplementary file 1 — Additional file 1: Table S1. Induced fit docking interactions for the reference ligand (6TC) and the selected top five hits. Table S2. Interactions between the examined ligands (6TC and the selected top five hits) and the UPPS protein during the simulation run. [file 13065_2023_1110_MOESM1_ESM.docx]

**Table S1. Induced fit docking interactions for the reference ligand (6TC) and the selected top five hits.**

| compound | IFD Interactions |
| --- | --- |
| Reference (6TC) | 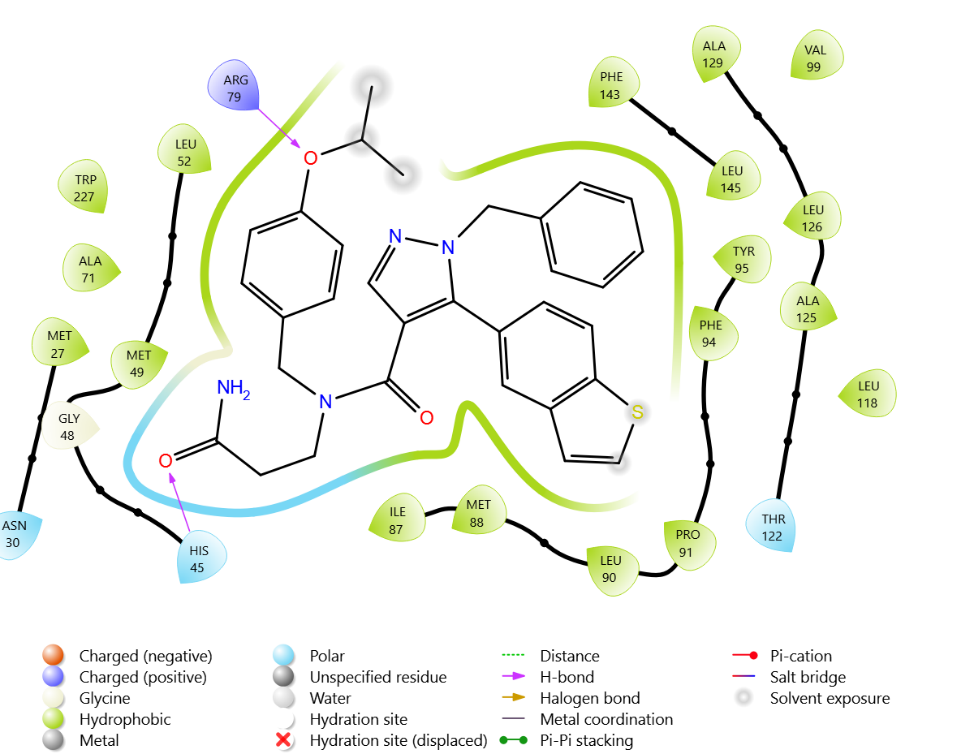 |
| CDI484583 | 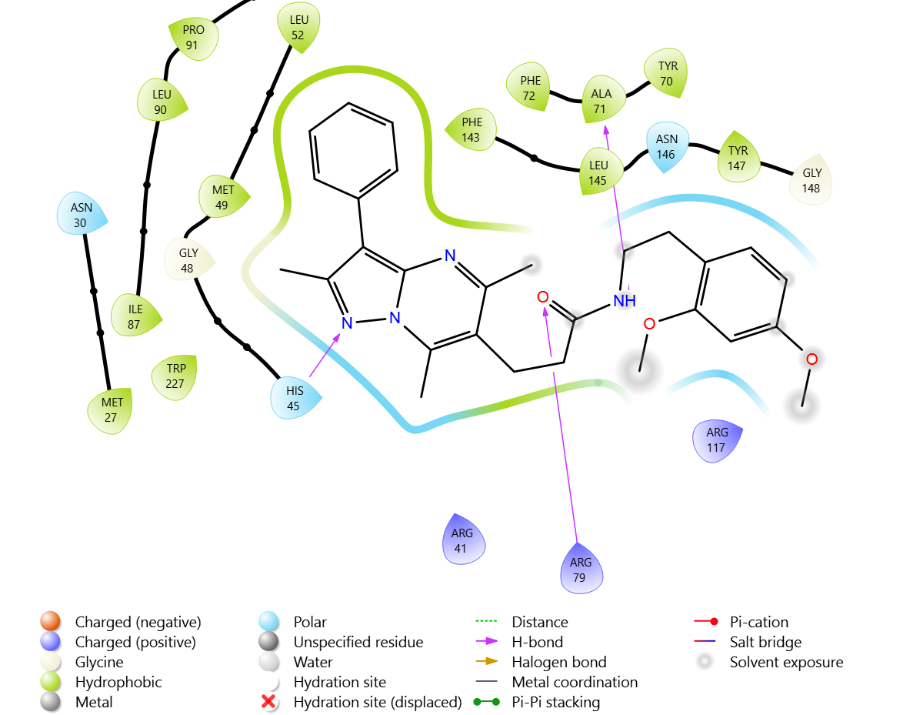 |
| ENA153723 | 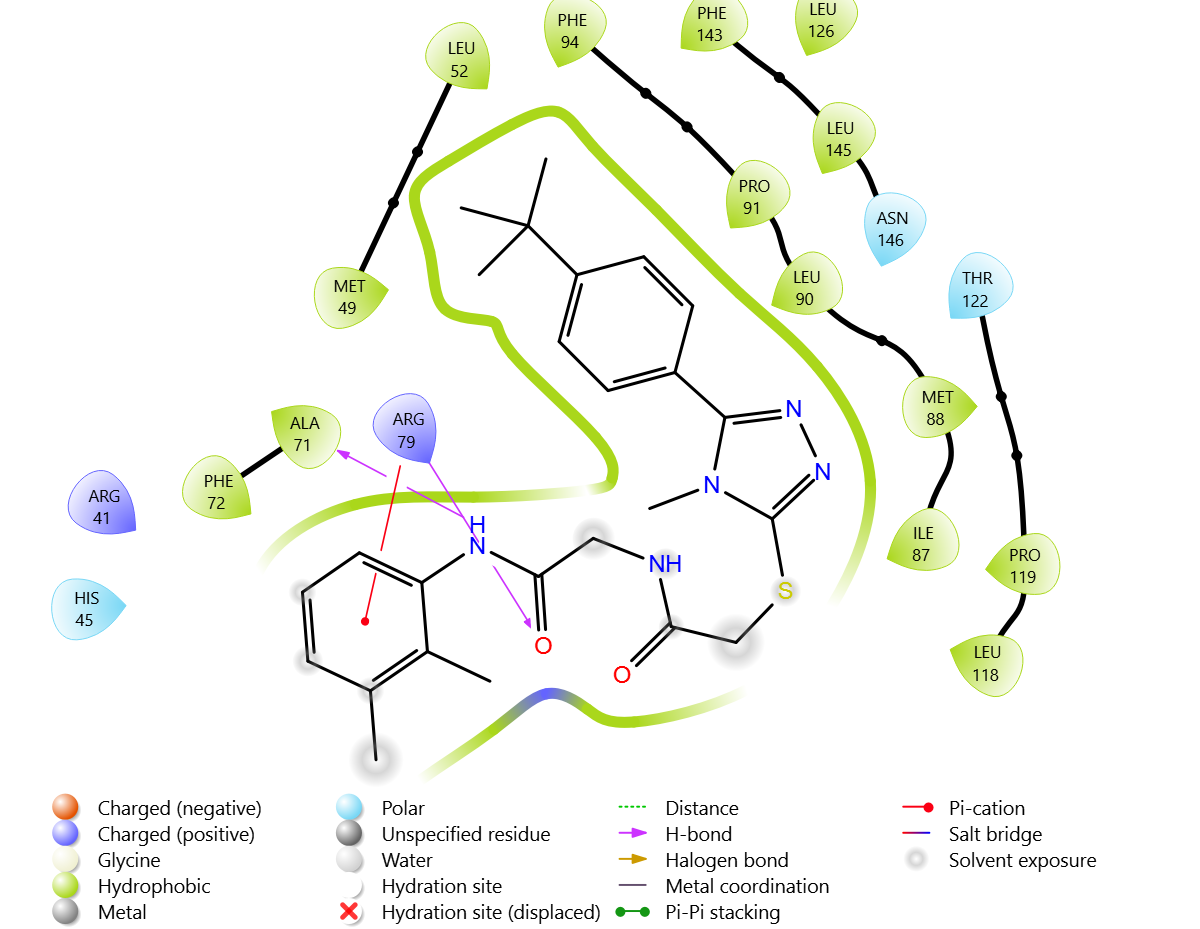 |
| 3lp2_LP9 | 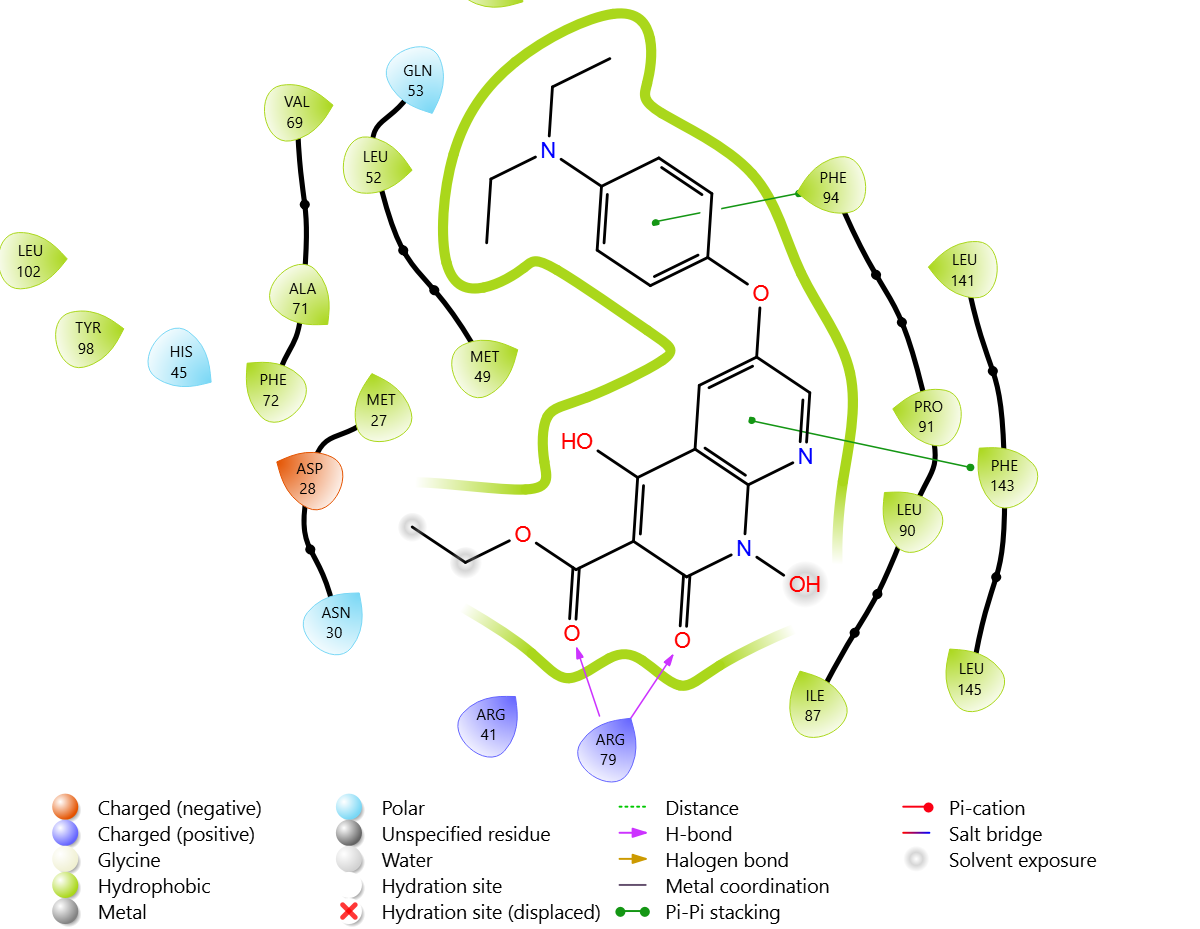 |
| ZINC  (000003986735) | 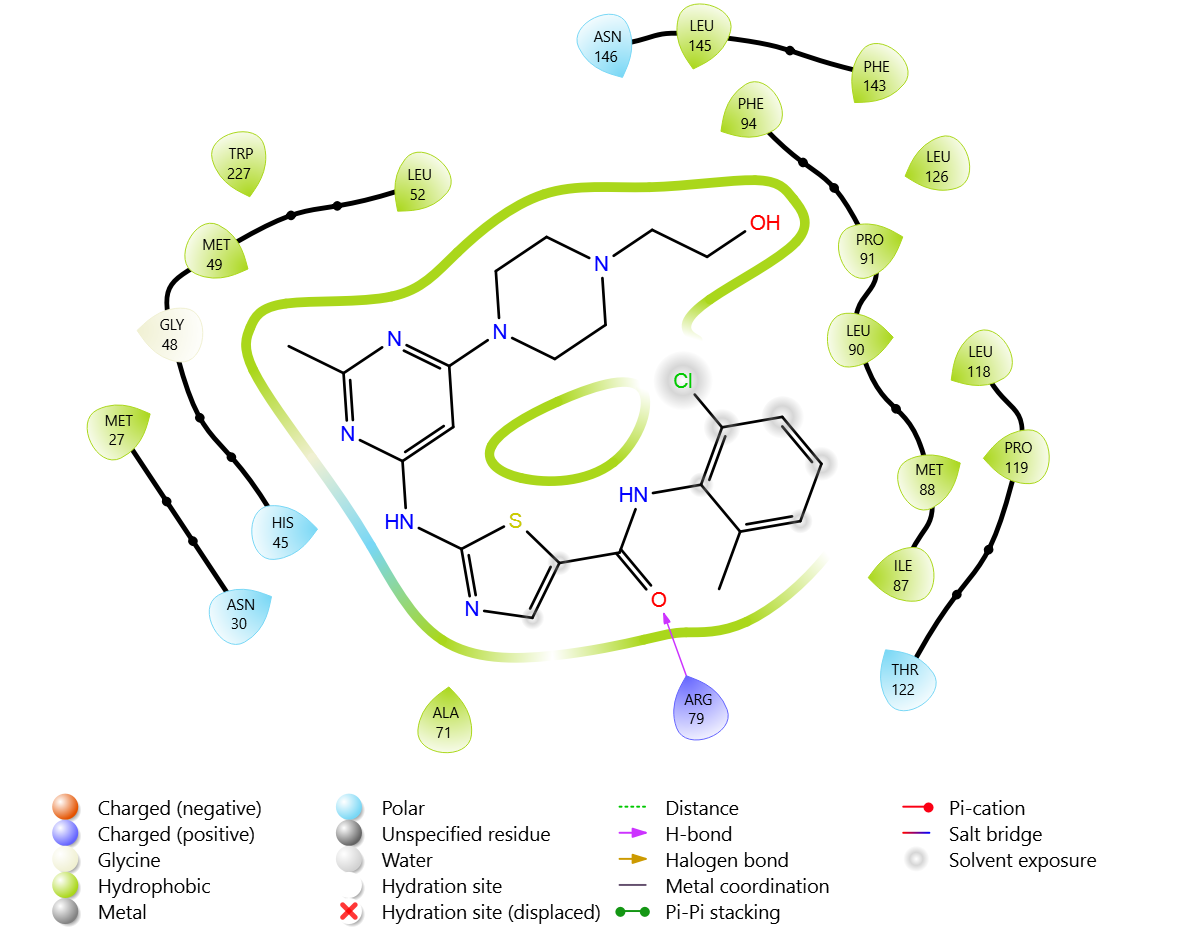 |
| Compound13509 | 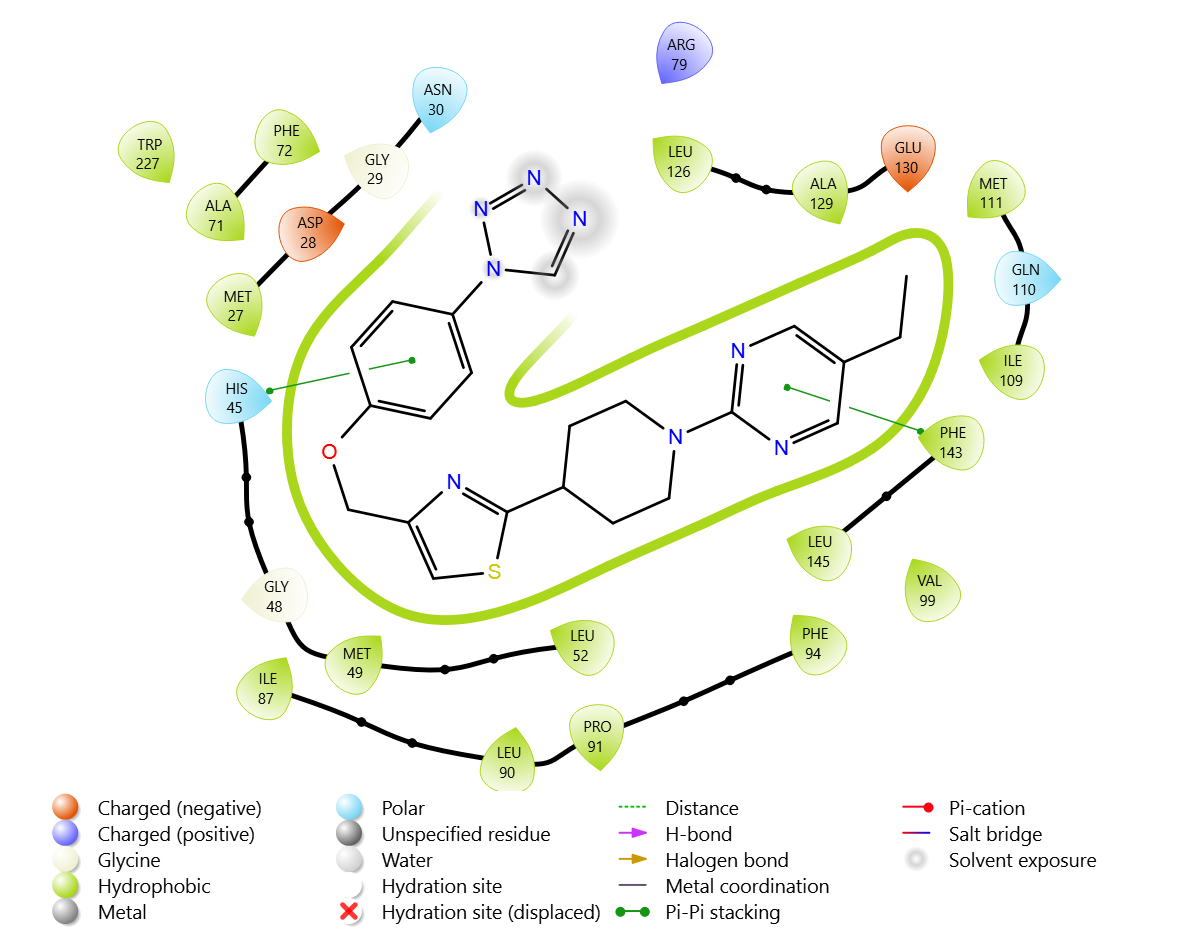 |

**Table S2. Interactions between the examined ligands (6TC and the selected top five hits) and the UPPS protein during the simulation run.**

| Compound | Interactions obtained from the MD run (100 ns) |
| --- | --- |
| Reference (6TC) | 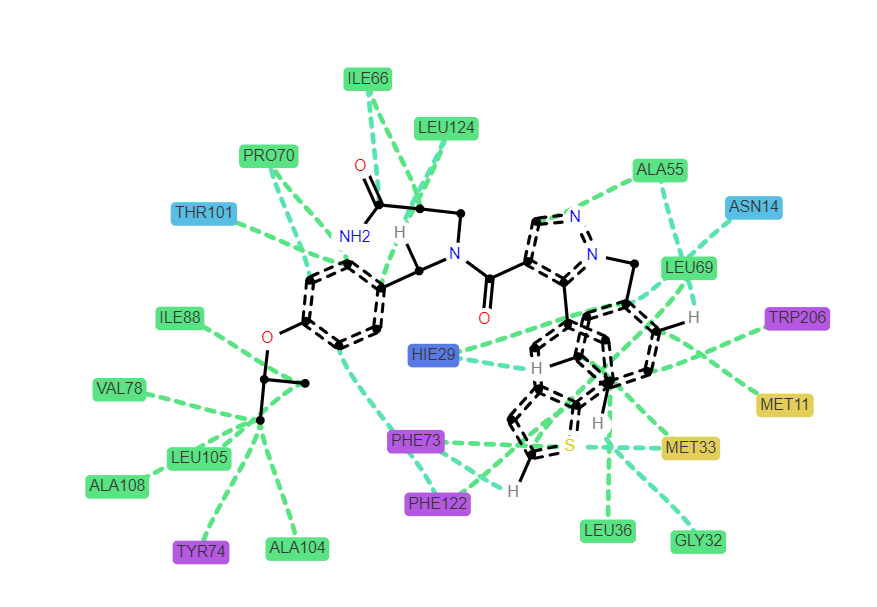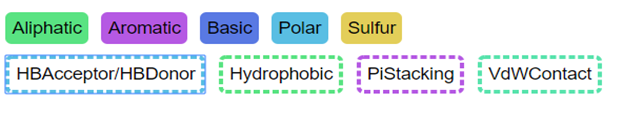 |
| CDI484583 | 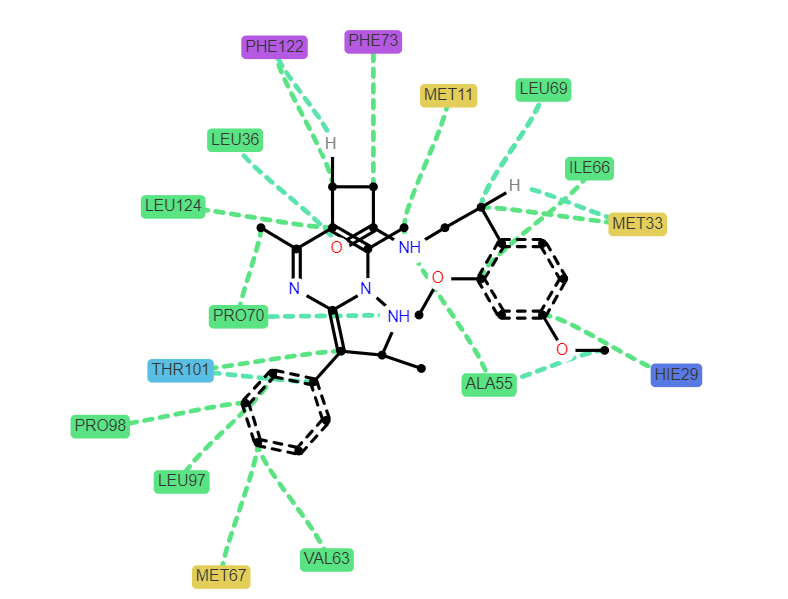  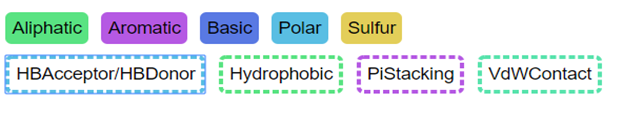 |
| ENA153723 | 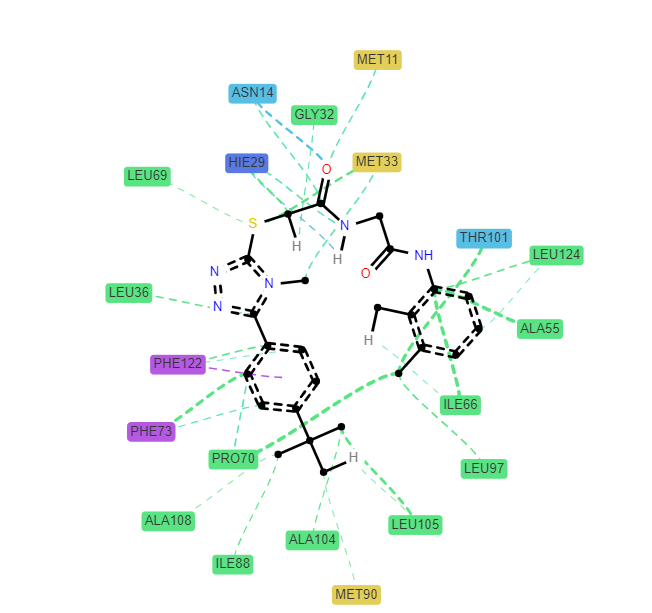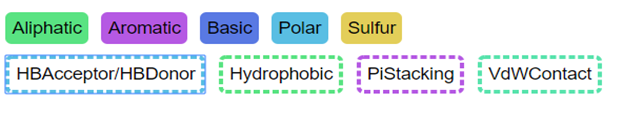 |
| 3lp2_LP9 | 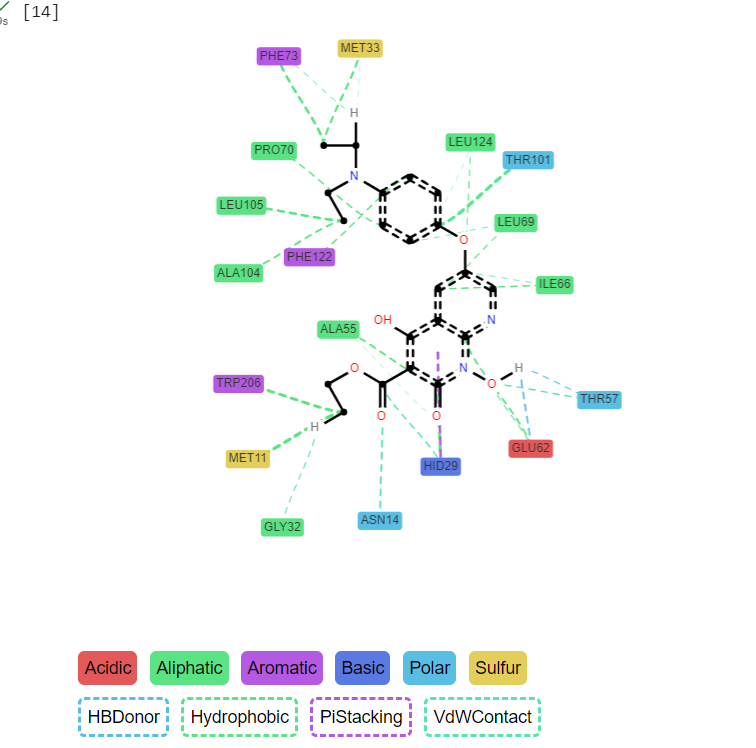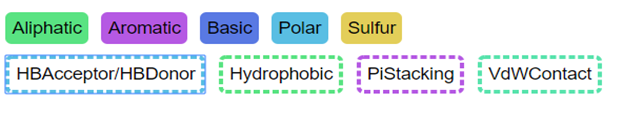 |
| ZINC  (000003986735) | 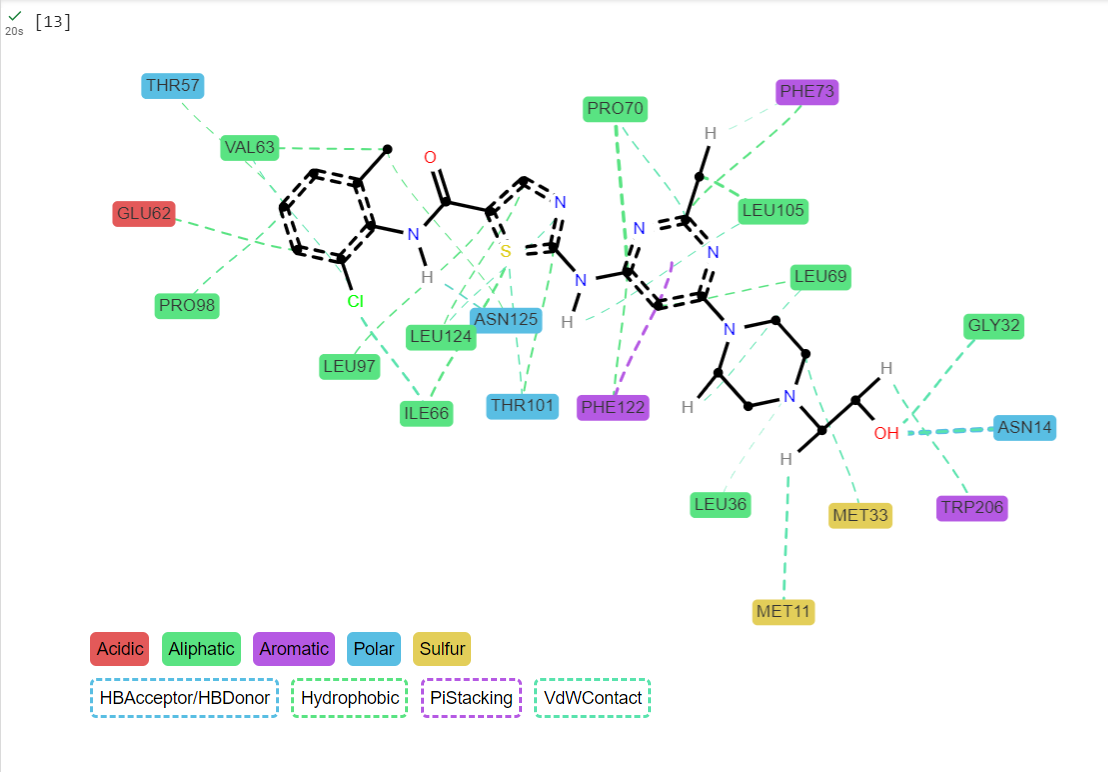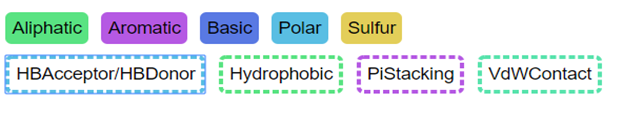 |
| Compound13509 | 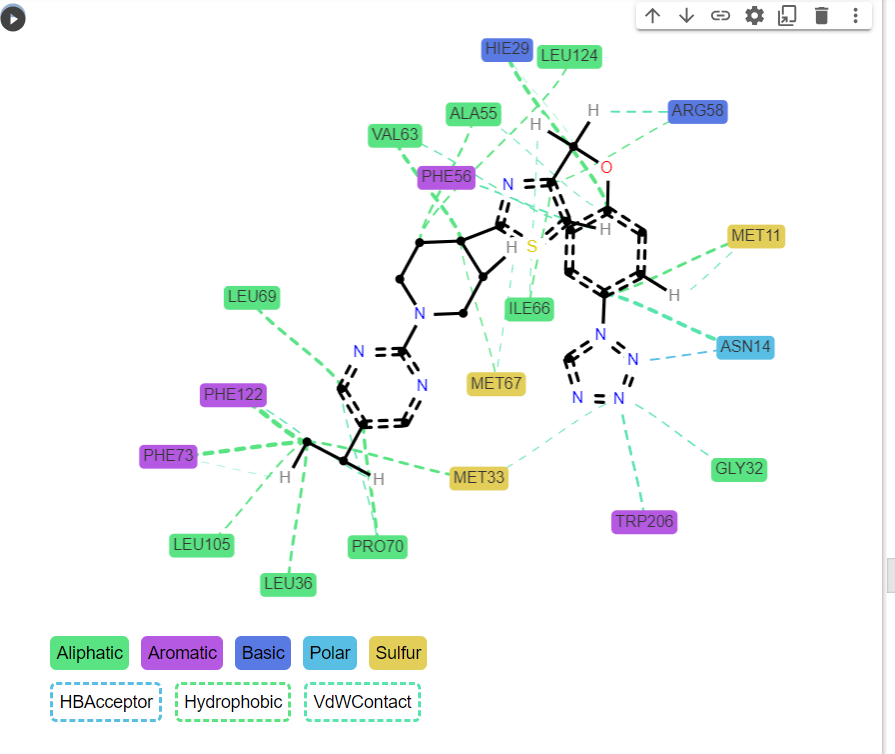  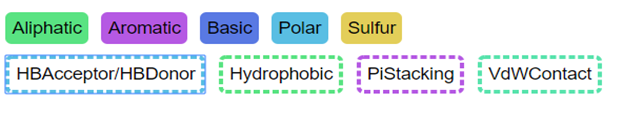 |
